# The Implication of the Gut Microbiome in Heart Failure

**DOI:** 10.3390/cells12081158

**Published:** 2023-04-14

**Authors:** Vasile Valeriu Lupu, Anca Adam Raileanu, Cristina Maria Mihai, Ionela Daniela Morariu, Ancuta Lupu, Iuliana Magdalena Starcea, Otilia Elena Frasinariu, Adriana Mocanu, Felicia Dragan, Silvia Fotea

**Affiliations:** 1 Faculty of General Medicine, “Grigore T. Popa” University of Medicine and Pharmacy, 700115 Iasi, Romaniamagdabirm@yahoo.com (I.M.S.);; 2 Faculty of General Medicine, Ovidius University, 900470 Constanta, Romania; 3 Faculty of Pharmacy, “Grigore T. Popa” University of Medicine and Pharmacy, 700115 Iasi, Romania; 4 Faculty of Medicine and Pharmacy, University of Oradea, 410087 Oradea, Romania; 5 Medical Department, Faculty of Medicine and Pharmacy, “Dunarea de Jos” University of Galati, 800008 Galati, Romania

**Keywords:** heart failure, gut microbiota, metabolites, dysbiosis, immune modulation

## Abstract

Heart failure is a worldwide health problem with important consequences for the overall wellbeing of affected individuals as well as for the healthcare system. Over recent decades, numerous pieces of evidence have demonstrated that the associated gut microbiota represent an important component of human physiology and metabolic homeostasis, and can affect one’s state of health or disease directly, or through their derived metabolites. The recent advances in human microbiome studies shed light on the relationship between the gut microbiota and the cardiovascular system, revealing its contribution to the development of heart failure-associated dysbiosis. HF has been linked to gut dysbiosis, low bacterial diversity, intestinal overgrowth of potentially pathogenic bacteria and a decrease in short chain fatty acids-producing bacteria. An increased intestinal permeability allowing microbial translocation and the passage of bacterial-derived metabolites into the bloodstream is associated with HF progression. A more insightful understanding of the interactions between the human gut microbiome, HF and the associated risk factors is mandatory for optimizing therapeutic strategies based on microbiota modulation and offering individualized treatment. The purpose of this review is to summarize the available data regarding the influence of gut bacterial communities and their derived metabolites on HF, in order to obtain a better understanding of this multi-layered complex relationship.

## 1. Introduction

Heart failure (HF) represents a worldwide health problem with significant associated healthcare costs, morbidity and mortality [1]. It is the final stage that results from cardiac structural and functional damage and subsequent imbalances in the compensatory mechanisms and pathogenic processes [2]. HF can take an acute form, correlated with several inflammatory markers and can also appears as a chronic disease, characterized by an altered inflammatory status associated with pro-inflammatory mediators that are considered essential in HF pathogenesis [3].

The bidirectional communication between gut microbiota and extra-intestinal organs has been intensively studied during the last two decades, leading to a better comprehension of the pathophysiological underlying mechanisms and offering a new characterization of HF clinical features, novel risk factors to be taken into account, new diagnostic tools and new therapeutic options [4,5,6]. A recent bibliometric study of Wu and colleagues [7], investigating the relationship between the human gut microbiota and heart failure, identified a large number of 873 literature studies, published from 2006 to 2021 and indexed by SCIE. This included original articles in a proportion of 81.79% and 18.21% reviews, with an additional number of 273 articles published between 1 January 2022 and 28 February 2023, identified by using a search query similar to the one used by Wu and colleagues [7]. The large number of scientific papers comes as proof of the researchers’ extensive efforts to understand the relationship between HF and the GI microbiota [7]. However, most studies available regarding this medical dyad are associative in nature and the subject clearly requires further investigation [8,9,10,11,12]. The aim of this review is to analyze the current evidence available in the literature regarding gut–heart interactions and the insights of the “gut hypothesis” of HF, highlighting the importance of the gut microbiota and their derived metabolites as a new frontier in HF research and a potential treatment target.

## 2. Gut-Associated Microbiome Composition and Function in Healthy Individuals

The human gut microbiome is considered to be an organ on its own with major interactions within the human organism, playing an active role in various immunological, neuronal, metabolic and endocrine responses [13]. The highest concentration and diversity of microorganisms from the human body lies in the gastrointestinal tract, consisting of more than 500 distinct species of bacteria, viruses, fungi and protozoa [14,15]. The GI microbiota are represented by five primary bacterial phyla: the *Firmicutes* (synonym *Bacillota*) and *Bacteroides* (synonym *Bacteroidota*) phyla predominate the microbiome and represent more than 90% of total bacterial communities, while the *Proteobacteria* (synonym *Pseudomonadota)*, *Actinobacteria* (synonym *Actinomycetota*), and *Verrucomicrobia* phyla are represented in smaller proportions [13,16]. Although the *Bacillota* phylum consists of more than 200 different genera such as *Bacillus*, *Lactobacillus*, *Enterococcus*, *Ruminococcus* and *Clostridium*, and the *Clostridium* genus represents 95% of the phylum. The *Bacteroidota* phylum is predominated by the *Prevotella* and *Bacteroides* genera. The *Actinomycetota* phylum is significantly less abundant than *Bacteroidota* phylum and the *Bifidobacterium* genus is its main representative [17]. Table 1 illustrates examples of taxonomic human gut microbial communities.

The microbiome is not inherited, but acquired, and its composition is changing through different stages of each individual’s life, with a unique composition and microbial diversity [19,20]. Its development starts early, in prenatal life, and continues during birth and through senescence [21,22]. The following interfere with microbiome composition, leading the way to health or disease: sex; genetics; the mother’s influence during pregnancy and birth; feeding practices in early childhood; dietary habits; antibiotics; tobacco and alcohol use; a sedentary lifestyle associated with the socioeconomic conditions; household pets; pollution; and geographical distribution [15,21,22,23,24]. 

Whereas each individual’s gut microbiota are characterized by a specific combination of bacterial species, due to inter-individual and intra-individual variations throughout human life, the human gut microbiota’s functions are highly preserved between individuals [13]. In addition to one’s genetic susceptibility, the diversity of the microbiome’s composition plays a key role in each individual’s personalized response to different environmental exposures such as diet, xenobiotics and medical treatments [2].

The GI mucosa represents the site of the human–external environment interaction. The GI microbiota and the intestinal barrier have bidirectional communication and form a complex network influencing the human state of health and disease [14]. Besides its function as an organ used for digestion and absorption, the GI tract acts as an immune organ, the human body’s largest immune organ [25]. “Healthy” gut microbiota have the capacity for: preserving the stability of the intestinal wall and its barrier function; tight epithelial junctions and a normal mucosal immunity; and preventing pathogen proliferation [2]. The gut-associated microbiota can regulate the inflammatory response directly, inducing either innate or adaptive immune responses, or it can alter the immune cells’ function using active metabolites, including short-chain fatty acids (SCFAs), trimethylamine N-oxide (TMA-O) and indoleacetic acid (IAA) [26,27,28]. It appears that dysbiosis of the gut bacterial communities produces alterations to the microecological environment of the gastrointestinal tract, becoming a pathogenic factor in a wide spectrum of diseases, including gastrointestinal disorders, inflammatory, respiratory, metabolic, and neurologic diseases [2,13].

Although for a long period of time microbiome study was based mainly on culture-based approaches, currently 16S rRNA gene sequencing is used for taxonomic classification of microbial communities. In addition, for the identification of potential metabolic functions of the associated microbiota, whole metagenome shotgun sequencing—which is required for the identification of potential metabolic functions of the associated microbiota—is now available, allowing in-depth study [29,30].

## 3. Impaired Gut Barrier Function and Inflammation in Heart Failure

**Figure 1:**
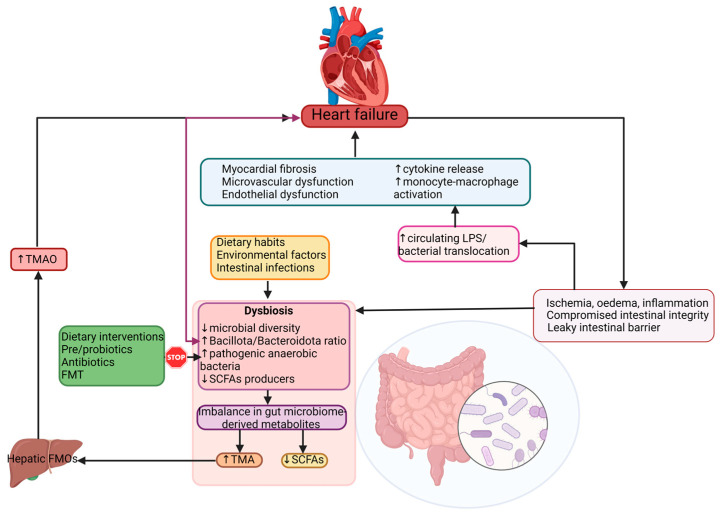
Concept of the gut–heart axis adapted to HF.

The “gut hypothesis” in HF suggests that there is a strong relationship between the gut microbiota, its metabolites and HF pathogenesis, as illustrated in Figure 1 [2,31]. Although this bidirectional communication is not fully understood, evidence indicates that this bacterial translocation appears in HF as a consequence of various mechanisms leading to structural and functional alterations of the GI tract, from splanchnic congestion to the host’s immunological defense system [7].

Available data suggest that the alteration of the structure and the function of the gut comes as a consequence of the microcirculatory perturbation that appears in HF patients [8]. In these patients, mainly in a decompensated form of the disease, the disruption of the normal composition of the gut microbial communities comes as a result of the intestinal hypoperfusion, that leads to changes in local pH and gut luminal hypoxia [32]. There is evidence of a disrupted intestinal epithelial function associated with HF: an alteration that seems to be the result of a reduced intestinal perfusion and ischemia [20,33]. A decreased cardiac output leads to an adaptive re-distribution of the systemic circulation to several end-organs [2]. Consequently, there appears to be an increase in intestinal wall edema, with the bowel wall thickening being positively related to increased markers of intestinal permeability, blood leukocytes and circulating levels of C-reactive protein [9].

Besides intestinal wall edema, HF is characterized by a reduction in the absorptive capacity and an increase in the epithelial permeability of the gut, facilitating the passage of several intestinal bacterial and/or endotoxins, such as lipopolysaccharides (LPS), from the gut to the systemic bloodstream [9,34]. LPS is a biologically active constituent of the Gram-negative bacterial wall with potential immunostimulatory activity by using the Toll-like receptor 4 (TLR4) pattern recognition receptor [35]. In HF patients, high LPS concentrations found in the hepatic veins sustain the hypothesis of an intestinal translocation process of gut microbes [36]. Moreover, it has been postulated that LPS itself can contribute to mucosal barrier functional deterioration, leading to HF progression [6]. 

The endotoxin intestinal absorption stimulates an increase in systemic inflammatory cytokines levels [20]. According to current data, HF appears to be correlated with a chronic state of inflammation that can be induced or accelerated by this microbial translocation, indirectly affecting cardiomyocytes’ normal function [37]. It seems that increased levels of circulating cytokines correspond to more severe clinical symptoms and to a worse prognosis in HF patients’ survival [38,39]. Serum levels of TNF-alpha, IL-1 and IL-6 of HF patients are directly influenced by the amount of existing LPSs, currently thought to be leading elements of a hyperinflammatory condition [25]. While in decompensated HF patients, LPS levels appear to be directly associated with systemic inflammation markers, and they decrease following HF recompensation. Treatment administration is not always followed by a decrease in plasma cytokine levels, suggesting a sustained effect as the disease progresses [20,40]. According to two large, randomized placebo-controlled trials, neither of the TNF-alpha antagonists’ administration decreased the risk of hospital admission or death in HF patients [41,42].

Another study in HF with reduced ejection fraction (HFrEF) patients with different stages of disease severity or with an advanced intervention, such as heart transplantation (HT) or a left ventricular assist device (LVAD), evaluated their blood and stool specimens. All subjects, from New York Heart Association (NYHA) Class I to IV, displayed an increase in their inflammatory marker levels. Following LVAD and HT, their levels decreased but failed to achieve normal values. LPS, however, augmented its levels across all NYHA classes and remained elevated in patients despite HT and LVAD intervention [43]. Similar to LPS, raised serum levels of IL-6, IL-1β and TNF alpha also induced intestinal permeability, promoting a vicious feedforward cycle of inflammatory cytokine augmentation and endotoxin translocation [44,45,46,47].

## 4. Dysbiosis in Heart Failure

Gut microbiota, as the most important active components in the intestinal microecosystem, have been shown to have a strong influence on HF. Besides the correlation with inflammation and increased intestinal permeability, an analysis using fluorescence in situ hybridization described the presence of bacterial overgrowth as mucosal biofilm and an increased bacterial adhesion in the sigmoid colon mucus of HF patients. The increased intestinal juxta mucosal bacterial biofilm has been correlated with an amplified immunoglobulin A–anti-LPS response [20,33].

In stable chronic HF with reduced ejection fraction (HFrEF) patients, an increased level of pathogenic bacteria such as *Salmonella*, *Shigella*, *Campylobacter* and *Yersinia* species, as well as yeasts including *Candida* species, have been reported as assessed by microbial culture methods; their levels being correlated with HF severity [10]. Consistent with these results, there is evidence that the *Escherichia*/*Shigella* genus is increased in the same patient known with HF, during its decompensated compared to the compensated phase of disease [48]. Indeed, pathogen overgrowth increases the risk of developing invasive gastrointestinal infections in HF patients. A U.S. nationwide study of hospitalized patients, treated with antibiotics, revealed that HF is more likely to develop an additional *Clostridium difficile* (*C. difficile*) infection and have substantially worse in-hospital prognosis, in comparison to non HF controls [49].

Using 16S rRNA gene sequencing Sun and colleagues [11] analyzed fecal samples of patients with severe forms of chronic HF and compared the results with the one obtained from healthy controls. They reported reduced alpha diversity in chronic HF patients and important differences in beta diversity between the two groups. *Bacillota* phylum was found to be dominating the chronic HF patient’s fecal microbiota, but in smaller levels than the controls. *Pseudomonadota* and *Actinomycetota*, however, were reported to be more abundant than in the control samples. Moreover, *Pseudomonadota* phylum was the second most abundant phylum found in severe chronic HF patients instead of *Bacteroidota* phylum. *Pseudomonadota* phylum is composed of Gram-negative bacteria, mainly pathogens, and is thought to be a microbial signature of dysbiosis in gut bacterial communities [11]. Zhang et al. [12] reported similar results, with reduced amounts of the *Bacillota* phylum and an augmentation in the *Bacteroidota* phylum [12]. At the genus level, the microbiota of chronic HF patients was found to be less abundant with *Faecalibacterium* and more abundant with *Escherichia*, *Shigella*, *Enterococcus* and *Klebsiella* spp. than that in the healthy controls [11]. The increased abundance of Gram-negative bacteria is responsible for the amount of LPS translocated into the bloodstream, accelerating HF progression [50].

A recent study by Zhang and colleagues [12] focused on patients with chronic HF: classes III and IV NYHA. They found important differences in alpha and beta diversity of the gut bacterial communities between the HF patients and controls. Moreover, they measured phenylacetylglutamine (PAGln), a metabolite produced by the intestinal microbiota, known to have higher plasma levels in patients known with major adverse cardiovascular events. They reported an increase in the PAGln concentration in HF patients in comparison to controls, and its levels increased with the severity of HF. Moreover, the authors reported the following: PAGln and brain natriuretic peptide (BNP), the most widely known bioactive hormone used for the diagnosis of HF, were negatively associated with *Bacteroides* and *Parabacteroides*; while *Romboutsia* and *Blautia* spp. were adversely correlated with PAGln; BNP was positively associated with *Klebsiella* spp.; BNP and PAGln were positively associated with *Shigella* and *Escherichia* ; *Alistipes* was not correlated with BNP, whereas *Parabacteroides* was negatively associated with the left ventricular end-diastolic diameter (LVEDD), a parameter known to reflect structural modifications in the left ventricle ejection fraction (LVEF) [12]. Since the basic pathological mechanisms of HF are represented by myocardial fibrosis and inflammation, these results fiercely sustain the strong association of gut microbiota dysbiosis, its derived metabolites and HF pathogenesis [23]. In HF patients, elevated PAGln levels could be used as indicators of renal dysfunction [51]. The levels of PAGln—which has also been shown to favor thrombosis—tracks with the NT-proBNP levels in chronic HF with reduced ejection fraction (HFpEF) patients, indicating a potential relationship between them [4,52].

Yuzefpolskaya and colleagues [43] have evaluated the microbiome of HF patients with different degrees of severity and reported that alpha diversity was reduced as disease severity levels increased and remained low despite receiving interventional treatment such as LVAD or HT, probably due to persistent inflammation. Alpha diversity seemed to be negatively correlated with levels of inflammation and endotoxinemia (LPS and sCD14). Therefore, as HF evolves into advanced stages, levels of endotoxinemia and systemic inflammation increase and the gut diversity of bacterial communities decreases [43]. Several studies of the intestinal bacterial profile in patients with acute decompensated or stable HFrEF have reported that HF patients have a significantly reduced alpha and beta diversity compared to healthy individuals, providing evidence for the HF-induced community composition shifting of the gut microbiota [5,13,53,54,55,56,57]. Similarly, another small study on HF patients reported a different microbiota composition between individuals with congestive heart failure and healthy volunteers, although between ischemic and dilated cardiomyopathy no noticeable differences could be identified [58].

In a much more comprehensive study, Jia et al. [59] reported elevated levels of several Streptococcus species and genera of the *Enterobacteriaceae* family, and a decreased abundance of *Faecalibacterium prausnitzii* and *Roseburia intestinalis*, known producers of the SCFA butyrate [60]. Zhu and colleagues [61] reported an elevated abundance of *Enterococcus*, *Escherichia* and *Shigella* spp. and a decreased abundance of *Roseburia*, *Faecalibacterium* and *Eubacterium rectale*, known as butyrate producers [61].

Kamo and colleagues [8] reported reduced diversity of microbial communities in the GI tract of HF patients, but also stated that HF-associated gut dysbiosis varied according to the patient’s age. Compared to younger patients known with HF, older patients seemed to display decreased levels of *Bacteroidota* and elevated amounts of *Pseudomonadota. Dorea longicatena and Eubacterium rectale*, members of the *Lachnospiraceae* family, were decreased in all patients known with HF while *Clostridium clostridioforme* and *Faecalibacterium prausnitzii*, members of the *Ruminoccaceae* family, were found in smaller amounts in older HF patients compared to younger patients [8].

*D. longicatena* is a bacterium that produces acetic acid, an SCFA, as a fermentation product. However, acetate can be further used as a substrate in order to generate butyrate [62]. *Eubacterium rectale*, another butyrate producer bacterium, was identified at increased levels in gut mucosal biofilms of HF patients by Sandek and colleagues [33]. In contrast, another study by Kamo et al. [8] reported decreased levels of the bacteria as characterizing HF [8]. *Faecalibacterium prausnitzii*, another butyrate-producing commensal bacteria with anti-inflammatory properties, was found to be decreased in abundance in HF patients, negatively affecting the intestinal permeability [58,63,64]. Butyrate-producing bacteria are essential for the state of well-being of each individual, as butyrate is used as an energy source for intestinal epithelial cells, and it regulates the integrity of the epithelial barrier and suppresses the intestinal and extra-intestinal inflammation [65,66]. The decreased levels of *F. prausnitzii* and increase in *Ruminococcus gnavus* were found to be important characteristics of gut microbiota in chronic HF patients [60].

Kummen and coleagues [5], in their two-cohort study, also identified a reduced diversity of the gut microbial communities with changes in fifteen core taxa. Moreover, they reported the depletion of the *Lachnospiraceae* family in HF patients, and found that it was inversely associated with soluble CD25 levels: that is a marker for T-cell and macrophage activation [5]. The reduced abundance of the *Ruminococcaceae* genera and *Lachnospiraceae* genera was also reported by other researchers [11,56,67]. As several members of *Lachnospiraceae* and *Ruminococcaceae* families are butyrate producers, the results of these reports drew attention to the microbial modulation of inflammation through its metabolites including short chain fatty acids (SCFAs). Cui and colleagues [58] also reported the depletion of SCFA-producing bacteria; *Ruminococcus* spp. in particular. Another commensal bacterium known to have a significant role in SFCA production is *Eubacterium hallii.* Similar to *Lachnospiraceae* family, their depletion in HF patients stool samples was correlated with an increased level of soluble CD25 and: furthermore, with death or hearth transplant [68].

Sun et al. [11] reported a reduction in the genus *Dialister* and an augmentation in the *Enterococcus* and *Enterococcaceae* genera, as notable features in chronic HF patients’ stool samples [11]. Luedde et al. [57], in their research work, discovered that HF patients’ GI microbiota were characterized by a significant reduction of the *Collinsella* and *Blautia* genera, together with two unknown genera from the *Ruminococcaceae* and *Erysipelotrichaceae* families. Furthermore, recent data from other inflammatory disease studies might lead to the conclusion that the depletion of these genera can support HF underlying mechanisms. *Collinsella* spp. has been linked to type 2 diabetes mellitus (T2DM) and systemic atherosclerosis. Interestingly, *Collinsella* was found in increased amounts in patients with atherosclerosis or T2DM, whereas Luedde and colleagues [57] reported that in HF patients *Collinsella* seemed to be depleted. Although abundant in atherosclerosis and T2DM, *Collinsella* appears to be found in lower amounts in HF patients with ischemic heart disease or DM, also, concluding that the depletion of *Collinsella* from the GI microbiota might be considered to be highly specific to HF [57,69]. In Table 2 are summarized the studies about the gut microbiota in patients with HF.

## 5. Risk Factors for HF and Gut Microbiota

It is known that people suffering from HF have various risk factors, but the majority of them has either hypertension, obesity, dyslipidemia, diabetes, is genetically predisposed to HF, is smoking, has a sedentary lifestyle or is making unhealthy dietary choices [73,74,75,76]. New evidence suggests that gut microbiota and its metabolites could have an impact on HF risk factors as well.

### 5.1. Dietary Choices

The Western diet (WD) is characterized by high sugar and refined carbohydrate intake with a high glycemic index; content that inhibits nitric oxide synthase, resulting in myocardial oxidative dysfunction, cardiac hypertrophy and cardiomyocyte remodeling, all known to be predisposing factors for HF [77]. This diet rich in fast-food aliments and glucose leads to dysbiosis state characterized by elevated *Pseudomonadota* and *Bacillota* levels, which increases the levels of TMAO and ceramides, promotes cholesterol accumulation in macrophages and promotes atherosclerosis development [78]. The WD also leads to lipid accumulation in the myocardium, chronic inflammation and obesity [79]. Increased levels of salt and dietary additives used in fast-food alimentary processing, including nitrites and phosphates, have been associated to an increased risk of HF. They alter the *Bacillota* to *Bacteroidota* ratio [80]. Moreover, this diet alters gut barrier permeability, characterized by the decreasing levels of *Bacteroidetes* spp., *Bifidobacterium* spp., *Clostridiales* spp., *Lactobacillus* spp. and *Akkermansia muciniphila*, as well as all gut barrier-promoting bacteria. Furthermore, the intestinal wall integrity seems to be disrupted by an increase in *Desulfovibrio* spp. and *Oscillibacter* spp. [80].

### 5.2. Obesity

Savji and colleagues [81] in their study reported that obesity and its associated dysmetabolism, including hyperlipidemia, hyperglycemia and insulin resistance, are strongly correlated with HF [81]. A pro-inflammatory environment characterized by elevated levels of pro-inflammatory cytokines is promoted by obesity and its associated cardiometabolic factors (insulin resistance, dyslipidemia and abdominal adiposity) [82]. The endothelial dysfunction and the nitric oxide unavailability might lead to left ventricular hypertrophy and systolic and diastolic dysfunction in HFpEF [82,83]. Furthermore, obesity can cause modifications in vasculature and blood volume which, associated to the increased consumption of oxygen, conducts to ventricular hypertrophy, increased mean pulmonary arterial pressure and elevated left ventricular diastolic pressure [84].

In both animal and human studies, obesity seems to be associated to a modified ratio between *Bacillota* and *Bacteroidota* phylum in most research, with a decrease in *Bacteroidota* and an increase in *Bacillota* [85]. The amount of *Bacteroidetes* found the intestinal microbiota has been reported to be relevant in obesity. Obese people that follow a calorie-restricted diet and lose weight seem to have an elevated ratio of *Bacteroidetes* species in their gut microbiota [86]. Specifically, *Clostridium bartlettii*, *Akkermansia muciniphila* and *Bifidobacteria*, all SCFA producers, have been negatively associated with obesity induced by a high fat diet and its metabolic complications [87,88].

### 5.3. Type II Diabetes Mellitus

Type II diabetes mellitus (T2DM) is a strongly associated risk factor for HF and other CVD. Patients known to have T2DM present a decreased level of bacterial genera such as *Faecalibacterium*, *Bifidobacterium*, *Akkermansia*, *Bacteroides* and *Roseburia. Roseburia*, *Bacteroides* and *Akkermansia* have anti-inflammatory effects. *Bacteroides* and *Akkermansia* in decreased levels lead to an under expression of tight junctions’ genes, elevated “leaky gut”, and, in consequence, endotoxemia [89]. Furthermore, the reduced abundance of the butyrate-producing *Faecalibacterium prausnitzii* and *Roseburia intestinalis* dysregulates the metabolism of fatty acids, leading to oxidative stress and its associated cardiometabolic adverse manifestations [90,91]. On the other hand, T2DM is positively associated with bacteria from *Fusobacterium* and *Ruminococcus* genera, and the phylum *Bacillota*, all with pro-inflammatory activity [92].

### 5.4. Hypertension

Persistently elevated blood pressure (BP) patients present a higher (up to five-fold) *Bacillota-*to-*Bacteroidota* ratio in comparison to normotensive controls [93]. Moreover, the intestinal microbiota is dominated by lactate-producing genera (e.g., *Turicibacter* and *Streptococcus*), while SCFA-producing ones appear to be reduced (such as *Clostridiaceae*, *Bacteroides and Akkermansia*) when hypertension is present [94,95]. Some of these associated perturbations in gut microbiota homeostasis are partially related to HF pathogenesis and increase the risk of HF progression.

## 6. Gut-Derived Metabolites as Possible Biomarkers Related to Intestinal Dysbiosis in HF

A biomarker is defined as a biological compound that is easily accessible and measurable in the body. Biomarkers can be classified as molecular, cellular or imaging. Their role is to help in identifying the disease or provide therapeutic guidance. Natriuretic Peptides (NP), brain-type natriuretic peptide (BNP), N-terminal prohormone of BNP and cardiac troponin measurements—classic HF biomarkers—have already been included in the guidelines for HF diagnosis and treatment by the European Society of Cardiology (ESC) [96] and the American Heart Association (AHA) [97]. The addition of other diagnostic and prognostic biomarkers that could be associated to such a complex disease would be of benefit for both patients and medical practitioners.

Gut microbial-derived metabolites can also play a significant role in the pathogenesis of HF. It appears that the gut microbiome acts similarly to an endocrine organ. By generating active biometabolites including short-chain fatty acids (SCFAs), trimethylamine (TMA)/trimethylamine N-oxide (TMAO), and bile acids, the gut microbiome influences the host physiology. Several studies described the association of the gut’s microbiome metabolites and different pathologies including hypertension, atherosclerosis, HF, obesity, chronic kidney disease, and T2DM [2,7,98,99,100,101]. These metabolites can be considered as biomarkers of intestinal dysbiosis and can predict inflammation in patients known with HF [101]. These patients with elevated plasma levels of phenylalanine display increased levels of inflammatory cytokines (IL-8, IL-10), C-reactive protein (CRP) and associate higher mortality [102], whereas glycine manifest anti-inflammatory effects and seem to offer protection to the cells and heart [103]. Furthermore, in an analysis of data gathered from the FINRISK and PROSPER cohorts, phenylalanine was reported to be an independent predictor of HF [104]. A recent study conducted by Hayashi and colleagues [105], used whole genome shotgun sequencing for analyzing fecal samples and mass spectrometry-based profiling of amino acids and identified a possible correlation between amino acid metabolic disturbances and gut dysbiosis in patients diagnosed with HF [105].

Alterations of gut microbiota composition, especially elevated N-oxidetrimethylamine (TMAO) levels are correlated with the risk of developing HF [106]. TMAO is a metabolite produced by gut bacteria including *Bacillota* and *Pseudomonadota*, obtained from choline, phosphatidylcholine, and L-carnitine fermentation [2]. Chen and colleagues [106] reported that an elevated level of TMAO resulted from a diet high in saturated fat and sugar can lead to fibrosis, myocardial inflammation and to impaired diastolic function [106]. Individuals with an increased abundance of *Ruminococcus*, *Prevotella* and *Clostridium* genera and the *Lachnospiraceae* family, and decreased levels of *Bacteroidota*, revealed higher levels of TMAO in their plasma [107,108]. HF–associated dysbiosis is characterized by high levels of circulating TMAO, that are able to stimulate cardiac remodeling through promoting myocardial fibrosis and pro-inflammatory effects [2,77,109,110]. Available evidence reports that the overexpression of cytokines with pro-inflammatory action, including Il-1β, and TNF-α and the attenuation of IL-10 and other cytokines with anti-inflammatory properties are both stimulated by increased levels of TMAO [78,111]. 

HF patients display elevated plasma levels of TMAO when compared to healthy individuals. Increased TMAO levels can be used as a prognostic biomarker in both acute and chronic HF, independently of B-type natriuretic peptide (BNP) and traditional risk factors, as TMAO levels are predictive of an augmented risk of mortality in these patients [80,81]. Elevated TMAO plasma values correspond with advanced stages of left ventricular diastolic dysfunction [80]. TMAO can also be considered a prognostic predictor of HFeEF and a marker of risk stratification for this particular category of patients [112,113]. As for hospitalized acute decompensated patients with HF, increased TMAO levels are correlated with a diminished renal function and can be used as a predictor of an elevated risk of death or readmission to hospital for a HF exacerbation [114]. Furthermore, TMAO level was also associated with hemoglobin, creatinine, BUN, and NT-proBNP [115]. Another study on gut-related metabolites with outcomes in HF reported that carnitine-related metabolites showed associations with adverse outcomes in acute HF, in particular L-carnitine and acetyl-L-carnitine for short-term outcomes (30 days after the acute event) and TMAO for long-term outcomes (1 year following the acute episode) [51].

The SCFAs are represented by acetate, propionate and butyrate, and they are generated by gut bacteria including *Bacteroides*, *Bifidobacterium* and *Faecalibacterium* spp. [53]. They are the most important metabolites produced through colon bacteria fermentation of resistant starch and dietary fibers [101]. Most evidence sustains the fact that SCFAs have a protective role against HF and play a major role in maintaining the integrity of the intestinal barrier: in mucus production and they are active in anti-inflammation protection [67]. However, increased SCFA levels in fecal samples are considered to be a marker of hypertension, central obesity and cardiometabolic disease subclinical measures [116]. There is evidence that SCFAs are closely associated to atherosclerosis [117]. In a rodent model, butyric acid supplementation through the diet inhibited the atherosclerotic lesions of apolipoprotein E (Apo-E) by reducing the macrophage migration rate, and increasing the collagen deposition and plaque stability [118].

In chronic HF patients, there was an increase in microbial genes responsible for LPS biosynthesis, lipid metabolism, tryptophan, and particularly TMAO production [58]. On the other hand, microbial genes for butyrate-acetoacetate CoA transferase, a vital enzyme for butyrate synthesis as well as SCFA-producing bacteria, were importantly reduced in chronic HF patients [119]. Levels of ricinoleic acid, a gut microbiota metabolite with anti-inflammatory proprieties, were found to be highly decreased in these patients’ plasma [119]. Moreover, ricinoleic acid levels were reported to be negatively associated with the bacterial communities found to be enriched in chronic HF patients’ guts and positively correlated to those dominating the microbiota of controls [58]. Elevated levels of cardiovascular-harmful metabolites including sphingosine 1-phosphate and a diminished value of beneficial cardiovascular metabolites such as orotic acid was also reported [58]. This functional alteration sustains the link between chronic HF and an imbalance of gut microbial communities and their metabolites.

Another group of researchers concentrated their efforts on a small group of elderly chronic HF patients. When evaluating the relationship between gut microbiota representatives and its metabolites, Wang et al. [71] reported that *Escherichia* and *Shigella* spp. were negatively associated with riboflavin and biocytin. *Haemophilus* spp. was negatively associated with cellobiose, alpha-lactose, lactose, isomaltose, sucrose, melibiose, turanose and trehalose. *Klebsiella* spp. was positively associated with ethylsalicylate and bilirubin, and negatively related to hexanoylcarnitine, citramalate, isovalerylcarnitine, inosine, methylmalonate and riboflavin. The authors concluded that the gut microbiota alteration in chronic HF is associated with various modifications of the serum metabolic map [71].

Luo et al. [120], in a two-sample mendelian randomized study, demonstrated that *Candida*, *Campylobacter* and *Shigella* spp were not correlated with an increased incidence of HF. However, when analyzing the genetic prediction it was suggerated that for every 1 unit increase in *Shigella* concentration, there is an increase of 38.1% in the relative risk for myocarditis and an increase of 13.3% for hypertrophic cardiomyopathy. Moreover, for every 1 unit increase in *Candida* concentration, there is an increase of 7.1% in the relative risk of chronic kidney disease. As for intestinal metabolites, the genetic prediction report suggested that the relative risk of myocardial infarction and HF increases by 1.4% and 1.7% separately, for every 1 unit increase in betaine [120].

## 7. Interactions between the Gut Microbiome and Cardiovascular Drugs

Age, sex, nutritional status, disease states, along with genetic and environmental exposures are factors that can explain how individuals will respond to drug therapies [121]. The human microbiome is known for its involvement in drug metabolism and pharmacological efficacy, but among them there is bidirectional communication, as drugs can also influence microbiota composition. 

Drug absorption is an elaborate process, depending on many factors such as their solubility and stability in GI fluids, their pH, GI transit period, permeability through epithelial membranes and the drugs’ interaction with the host and microbial enzymes [122]. The human gut microbiota is genetically capable of producing enzymes involved in oral drugs’ metabolism, facilitating their absorption across the gut and through the bloodstream [121]. Dysbiosis of the gut’s bacterial communities can further alter drug pharmacokinetics; the activation of prodrugs can contribute to the production of unwanted toxic metabolites and the inactivation of drugs [123]. Variation in drug response can also be present in a “healthy” gut, due to inter-individual differences in intestinal bacterial species [13]. 

Related to the cardiovascular medication used in HF patients, metagenomic sequencing of stool samples from HF patients revealed that the use of several pharmaceutical agents such as statins, beta-blockers, angiotensin-converting enzyme inhibitors and platelet aggregation inhibitors has an important influence on gut microbial composition [124]. Despite the fact that specific underlying mechanisms are unknown, partial results of this study were reproduced by another British group of researchers [125]. Examples of microbial biotransformations are listed in Table 3.

### 7.1. Cardiac Glycosides

Digoxin, a drug frequently recommended in HF is a good example of microbiota influencing drug bioavailability. Some strains of *Eggerthella lenta* are responsible for converting digoxin into an inactive microbial metabolite, limiting the quantity of active drug absorbed into the systemic bloodstream in an important 10 percent of patients [126,133]. Recent studies offered proof that coadministration of digoxin together with antibiotics or an arginine rich diet both resulted in elevated systemic digoxin levels and clinically relevant fluctuations in drug levels [126,134].

### 7.2. Blood Thinners and Gut Microbiota

Aspirin is a non-steroidal anti-inflammatory drug (NSAIDS) commonly used to decrease the risk of cerebrovascular and cardiovascular disorders [133]. Existing evidence demonstrates its ability to disrupt the gut’s microbiota composition. Patients using aspirin present variations of *Ruminococcaceae*, *Prevotella*, *Barnesiella* and *Bacteroides* bacterial levels in comparison to individuals not using or using other types of NSAIDs. Furthermore, the gut’s bacterial communities’ composition seems to exert influence on aspirin metabolism. While oral antibiotic administration can decrease the gut microbiota’s metabolic activity by slowing its degradation, increasing its bioavailability and prolonging its anti-thrombotic action, probiotics containing *Bifidobacterium breve* Bif195 bacteria can protect against an aspirin intake adverse reaction, such as intestinal wall damage and aspirin-induced gastric ulcers [135,136].

Warfarin, a frequently used anticoagulant expresses its effect by inhibiting vitamin K-dependent activation of clotting factors II, VII, IX and X. Bleeding episodes associated with warfarin use increased when given together with antibiotics [137]. Two mechanisms have been cited. Antibiotics can interfere with warfarin use through inhibition or induction of CYP enzymes and can also alter the intestinal bacterial composition, eliminating vitamin K-producing bacteria, such as the *Bacteroides* genus [132,138].

### 7.3. The Effects of Beta-Blockers, ACEi, and ARBs on Gut Microbiota

The effects of antihypertensive medications have been investigated on several occasions, both in animal and human studies. Despite expectations, the association between the use of beta-blockers, angiotensin receptor blockers (ARBs) and angiotensin converting enzyme inhibitors (ACE inhibitors) can modify the composition of gut microbiota. A positive association was reported from a large metagenomics study, between calcium channel blockers, ACE inhibitors and bacterial composition of the gut [139]. Moreover, ACE inhibitors, including captopril, have been shown to have beneficial effects on hypertensive rats by diminishing gut dysbiosis, ameliorating the intestinal wall’s permeability and increasing villi length [121,131].

### 7.4. Statins and Gut Microbiota

Statins are drugs used for their capacity to decrease low-density-lipoprotein-C (LDL-C) and cholesterol levels. Inter-individual variations in the response to statin treatment are well-known, and are not related to a specific statin agent or dose [124]. Studies have proven their action on modulating gut bacterial communities’ composition [121,128]. Individuals treated with atorvastatin presented an increased level of anti-inflammatory gut bacteria such as *Faecalibacterium prausnitzii* and *Akermansia muciniphila*, whereas untreated patients known with hypercholesterolemia displayed an increased level of bacterial species with pro-inflammatory effects, such as *Collinsella* and *Streptococcus* [140]. LDL-C levels seem to be negatively correlated to the phyla *Bacillota* and *Fusobacteria*, while *Lentisphaerae* and *Cyanobacteria* spp. were positively associated with LDL-C [128]. Existing evidence suggests that the LDL-C response to statin treatment can be influenced by bacteria containing bile salt hydrolases (bsh). Administration of *Lactobacillus reuteri*, one of the gut’s bacteria with elevated bsh activity, resulted in an important reduction of LDL-C levels [141]. The same study reported that individual variations in LDL-C levels were inversely correlated with circulating bile acids. The *Bacillota* phylum, previous negatively associated with LDL-C levels, has also been associated with bsh activity [128,142]. Several animal models sustain the beneficial effect of statin therapy on microbial communities of the gut [129,140,143].

However, an uncommon side effect has been reported with rosuvastatin use. Due to a tertiary amine contained in rosuvastatin that competes with TMA for metabolism at the liver level, there is an increase in serum TMA levels and its excretion in urine, resulting in fish odor syndrome [144].

## 8. Modulation of Dysbiosis as a Potential Target in Heart Failure

Considering that dysbiosis is a key factor in HF pathogenesis and disease progression, targeting the disrupted gut microbiota could be considered an effective therapeutic objective. The possibility of characterizing each patient’s gut microbiota and his disease-associated dysbiosis allows the initiation of a personalized, targeted therapeutic plan. Although there are various ways to manage and modulate the dysbiotic intestinal microbiota, such as dietary interventions (which also include the use of prebiotics, prebiotics and postbiotics) and fecal transplantation, several reports from the available literature place diet modification and probiotic use as the main interventions for microbiota modulation.

Diet has always been considered a crucial factor in shaping the structure and function of gut’s associated microbiota. A 5-day adjusted diet has been shown to produce beneficial changes in the number and species of the gut microbiota [145]. Often cited in the medical literature, the Mediterranean diet (MD) consists of elevated levels of polyunsaturated fatty acids, dietary fiber, polyphenols, and a small quantity of red meat [77]. Among its recognized benefits on human health, an MD provides an increased abundance of probiotics, greater biodiversity, elevated SCFAs, and reduced TMAO [146,147]. Adherence to an MD was associated with a decreasing HF incidence up to 74% [148]. Moreover, it seems that the high compliance to MD is negatively associated with HF and improved the long-term prognosis for HFpEF patients, as it results from a 10-year follow-up. The MD might have an anti-inflammatory effect, as the beneficial action correlates with CRP levels [149,150]. The Dietary Approaches to Stop Hypertension (DASH) eating plan represents a diet that is rich in whole grain aliments, vegetables, fruit and low-fat dairy foods, and offers a significant potential in decreasing the HF incidence [151,152].

A high-fiber diet has recently been demonstrated to improve gut dysbiosis (described by the *Bacilliota* and *Bacteroidota* ratio), reduced blood pressure, improved cardiac function and normalized cardiac hypertrophy in a hypertension-induced HF experimental model [153]. Additionally, fermentation of fiber results in augmented SCFA production, with their beneficial actions on human health [150].

The World Health Organization defines probiotics as living microorganisms that have positive effects on the host when given in the right amounts [154]. Among their beneficial effects, we recall their capacity of regulating the altered intestinal microbiota, the protection of the integrity of the epithelial barrier, their capacity to inhibit the adhesion of pathogenic microbiota through competition, their encouragement of the production of B-cell-secreting IgA, mucin, as well as SCFAs with immune modeling and anti-inflammatory effects [154,155,156,157]. The most used probiotics are different strains of bifidobacteria, yeasts, and lactic acid bacteria [40,59].

In a rat model, oral administration of *Lactobacillus plantarum 299v* and *Lactobacillus rhamnosus GR-1* induced beneficial cardiac effects [158,159,160]. Lactobacillus supplementation seems to promote SCFA-producing bacteria such as *Eubacterium*, *Roseburia* and *Ruminococcus* in order to facilitate the dietary fiber-fermented byproduct SCFA, with critical roles in maintaining a healthy cardiovascular activity [161,162]. Although most studies on probiotic administration efficacy in HF are in animal models, there have also been a few reports describing clinical improvement by gut microbiota-mediated therapy in patients with HF [163]. In a small double-blind, placebo-controlled pilot study on HF patients (NYHA class II or III, with LVEF < 50%), were randomized to probiotic treatment receiving *Saccharomyces boulardii* (1000 mg per day for 3 months) or placebo. HF patients that followed probiotic treatment showed a reduction in total cholesterol levels and in uric acid levels, reporting an improvement in cardiac systolic function when compared with the placebo group [164]. Another three months’ treatment with rifaximin or *S. boulardii* reported no clinically significant effect on LVEF, circulating levels of TMAO, microbiota diversity and function or systemic inflammation in HF with reduced ejection fraction [165]. 

Related to antibiotic use in modulating the gut microbiota in HF patients, results are controversial. In animal models, oral vancomycin use induced smaller left ventricular infarct size, and improved recovery cardiac function following ischemia/reperfusion experiments in treated, compared to untreated, rats [166]. Rifamixin, besides its bactericidal and bacteriostatic effect, also has the capacity to reduce translocation of bacteria and toxicity, has an anti-inflammatory effect and can positively regulate the composition of the intestinal microbiota, promoting the growth of *lactobacillus* and bifidobacteria [167,168]. As for human clinical trials, the results are contradictory. The use of a cocktail of tobramicyn and polymixin B, in HF patients, normalized the level of intestinal Gram-negative bacilli, significantly decreased pro-inflammatory cytokines and improved flow-mediated dilation: evidence of endothelial dysfunction [169]. However, the results were limited to the treatment administration period. Furthermore, when prescribing an antibiotic therapy, side effects such as polymyxin B toxicity and macrolides’ increased risk of myocardial infarction must be considered [170]. 

A recent study that evaluated the effect of symbiotic administration on left ventricular hypertrophy and its effect on blood pressure and hsCRP as an inflammatory biomarker in chronic HF patients reported that, after 10 weeks of daily administration, the level of NT-proBNP, as a marker of left ventricular hypertrophy, decreased significantly in the symbiotic group compared to the placebo group. No significant differences were noted on hsCRP levels or blood pressure values [55].

Prebiotics are “a selectively fermented ingredient that results in specific changes in the composition and/or activity of the gastrointestinal microbiota, thus conferring benefit(s) upon host health” [171]. Prebiotics use could increase the amount of *Bifidobacterium* and promotes a higher body weight loss, which decreased systolic and diastolic blood pressure [172]. A recent study reported that prebiotic oligofructose reduces infiltration of inflammatory cells in rats [173]. Prebiotic administration can promote the development of beneficial bacteria, including *Bifidobacterium* and *Lactobacillus* spp, reducing body weight and inflammation and an improving glucose and insulin tolerance [174], all associated to better HF outcomes [75].

Regarding the regulation of the harmful metabolite production by the gut microbiota, preclinical studies reported beneficial effects of DMB administration as well as both dietary TMAO removal and administration of choline TMA lyase inhibitor, iodomethylcholine, in decreasing serum TMAO levels, ameliorating cardiac remodeling and reducing the expression of pro-inflammatory cytokines [175,176]. Resveratrol has also been shown to stimulate the growth of beneficial bacteria in the intestinal tract through the reconstitution of intestinal microflora, thus decreasing the production of TMAO [171].

Fecal microbiota transplantation (FMT) has been proven to be an effective method of reconstructing normal intestinal function and treating microecological imbalance in several disorders by introducing bacteria or metabolites from donor feces into diseased receptors [59,177,178,179,180]. A recent study reported that FMT and tributyrin treatment improved early cardiac dysfunction and increased the catabolism of branched chain amino acids in a diet-induced pre-HFpEF rodent model [15]. On human subjects, FMT normalized insulin sensitivity of obese individuals with metabolic syndrome, but the effects were short-term [181]. Currently, there are no clinical studies available to evaluate FMT outcome in HF patients, but FMT has great therapeutic potential and represents a promising direction for future research [101,182]. According to ClinicalTrials.gov there are four clinical trials focused on the efficacy and safety of different strategies regarding gut microbiota modulation in HF patients. Another 21 studies share the same objective but they include patients with different CVD: two of them evaluating the effect of gut microbiome restoration via FMT. 

The lack of uniformity in FMT’s clinical results and the increased efficiency associated with some particular strains dominating the donor’s fecal microbiota raise the question whether a more targeted therapy would have better results. A defined consortium of bacteria or single strains that would have been rationally selected based on their mechanism of action are already subjects of interest and promising results are expected to be published [15,50]. 

Until the present moment, several approaches such as the administration of antibiotics, probiotics, prebiotics, symbiotic and fecal microbiota transplantation have been tried to diminish the dysbiosis associated with HF, without any clear evidence of their efficacy and safety. These unsatisfying results might come as a result of these therapeutic approaches being highly generic and non-specific. Bearing in mind that each individual’s specific gut microbiota composition and the microbial signature associated with different disorders, medical providers must concentrate on designing personalized medicine that focuses on each individual’s characteristic gut microbiome features.

## 9. Limitations in the Study of Gut Microbiota and Their Implications in HF

Although an extensive characterization of the microbiome has been performed in recent years, there are still limitations that need to be overcome in gut microbiome study. Variation in study design as well as confounding variables in different studies frequently result in discordant results. The available experimental and bioinformatics methods leave space for bias and unreliable results [183]. There is also a lack of compatibility between existing databases, mainly because there is not a correct scale to be used when comparing the taxonomy and the functions associated with the human microbiome [184]. Currently, there are no quantitative definitions regarding microbial dysbiosis available, as this concept seems to be host-specific and disease-specific. There is a huge amount of data resulting from human microbiome studies and artificial intelligence techniques, although not available on a large scale, which would be useful after synthesizing them [185].

As for the research of the gut microbiome influence on HF, most studies are focused on bacterial communities while other members in gut microbiota such as virus, fungal, or archaea are not widely studied and thus their roles in human disease remain underappreciated. The exact microbial composition of HF patients in current studies is not the same and a common microbiome associated with HF has not yet been established. The great heterogeneity of HF populations has an important influence on common factors influencing microbiota composition. Another negative aspect would be that most of the existing research includes small size groups and the outcome is not adjusted for various factors and medications that may affect the growth of the gut bacteria, thus offering results with low statistical relevance. Moreover, HF risk factors vary depending on age, and the composition of gut microbiota and metabolites may also change with age [186]. Therefore, the critical role of age may affect the stability of the results.

## 10. Conclusions

The composition and function of the gut-associated microbiota and their pathophysiological role in human health have been active fields of research in recent decades. The continuous advance of modern technology pushes the frontiers of HF research further away, exploring new aspects of HF. The aim of this review was to summarize the available data regarding the influence of gut bacterial communities and their derived metabolites on HF and its associated risk factors. HF has been linked to gut dysbiosis, low bacterial diversity, intestinal overgrowth of potential pathogenic bacteria and a decrease in SCFA-producing bacteria. An increased intestinal permeability allowing microbial translocation and the passage of bacterially derived metabolites into the bloodstream is associated with HF progression.

Dysbiosis is a key factor in HF pathogenesis and disease evolution, and targeting the disrupted gut microbiota could be considered an effective therapeutic objective. There are many methods available in order to modulate the dysbiotic intestinal microbiota, such as dietary interventions (which include prebiotics, probiotics, and postbiotics) and fecal transplantation. Treatment results vary, however, as they highly depend on the baseline characteristics of each individual, including genetic background, gut barrier function and microbiome diversity. Development of personalized microbiome therapy, thus, is the key to successful clinical treatment in HF.

## Figures and Tables

**Table 1:** Main representatives for taxonomic gut microbial composition, adapted from Rinninella et al. [18].

Main GI PHYLUM	CLASS Examples	ORDER Examples	FAMILY Examples	GENUS Examples	SPECIES Examples
*Actinomycetota*	*Actinobacteria*	*Actinomycetales*	*Corynebacteriaceae*	*Corynebacterium*	
		*Bifidobacteriales*	*Bifidobacteriaceae*	*Bifidobacterium*	*Bifidobacterium longum* *Bifidobacterium bifidum*
	*Coriobacteria*	*Coriobacteriales*	*Coriobacteriaceae*	*Atopobium*	
*Bacillota*	*Clostridia*	*Clostridiales*	*Clostridiaceae*	*Faecalibacterium*	*Faecalibacterium prausnitzii*
				*Clostridium*	*Clostridum* spp.
			*Lachnospiraceae*	*Roseburia*	*Roseburia intestinalis*
			*Ruminococcaceae*	*Ruminococcus*	*Ruminococcus faecis*
	*Negativicutes*	*Veillonellales*	*Veillenellaceae*	*Dialister*	*Dialister invisus*
	*Bacili*	*Lactobacillales*	*Lactobacillaceae*	*Lactobacillus*	*Lactobacillus reuteri*
			*Enterococcaceae*	*Enterococcus*	*Enterococcus faecium*
		*Bacillales*	*Staphylocaccaceae*	*Staphylococcus*	*Staphylococcus leei*
*Bacteroidota*	*Sphingobacteria*	*Sphingobacteriales*	*Sphingobacteriaceae*	*Sphingobacterium*	
	*Bacteroidia*	*Bacteroidales*	*Bacteridaceae*	*Bacteroides*	*Bacteroides fragilis*
					*Bacteroides vulgatus*
					*Bacteroides uniformis*
			*Tannerellaceae*	*Tanarella*	
				*Parabacteroides*	*Parabacteroides diastonis*
			*Rikenellaceae*	*Alistipes*	*Alistipes finegolddi*
			*Prevotellaceae*	*Prevotella*	*Prevotella* spp.
*Pseudomonadota*	*Gamma proteobacteria*	*Enterobacterales*	*Enterobacteriaceae*	*Escherichia*	*Escherichia coli*
				*Shigella*	*Shigella flexneri*
	*Delta proteobacteria*	*Desulfovibrionales*	*Desulfovibrionaceae*	*Desulfovibrio*	*Desulfovibrio intestinalis*
				*Bilophila*	*Bilophila wadsworthia*
	*Epsilon proteobacteria*	*Campylobacterales*	*Helicobacteraceae*	*Helicobacter*	*Helicobacter pylori*
*Verrucomicrobia*	*Verrucomicrobiae*	*Verrucomicrobiales*	*Akkermansiaceae*	*Akkermansia*	*Akkermansia muciniphila*

**Table 2:** Gut microbiota composition studies in patients with heart failure (HF).

Study	Patients	Patients Age	Sample size	Method	Gut Microbiota Profile
Kamo et al. [8]	Acute HF or exacerbation of chronic HF	47.4 ± 2.8 years 73.8 ± 2.8 years	*n* = 12 HF < 60 years *n* = 10 HF > 60 years *n* = 12 controls	16S rRNA	↓ *Eubacterium rectale*, *Dorea longicatena* Depletion of *Faecalibacterium* in older patients
Sandek et al. [9]	Chronic HF	67 ± 2 years	*n* = 22 Chronic HF *n* = 22 control	Fluorescence in situ hybridization	↑ *Eubacterium rectale* *Faecalibacterium*
Pasini et al. [10]	Chronic HF	65 ± 1.2 years	*n* = 60 HF *n* = 20 control	Traditional culture techniques	↑ Campylobacter Shigella Salmonella Yersinia enterolytica Candida
Sun et al. [11]	Chronic HF	60.69 years	*n* = 29 HF *n* = 30 controls	16S rRNA	↓ *Ruminococcaceae* *Lachnospiraceae* *Dialister* *↑ Enterococcus* *Enterococcaceae*
Zhang et al. [12]	Chronic HF	65–86 years	*n* = 29 NYHA III HF *n* = 29 NYHA IV HF *n* = 22 controls	16S rRNA	↑ *Escherichia* and *Bifidobacterium* (NYHA III) ↑ *Klebsiella* and *Lactobacillus* (NYHA IV)
Luedde et al. [57]	Chronic HF: 70% exacerbation, 30% stable	65 ± 3.2 years	*n* = 20 HF *n* = 20 controls	16S rRNA	↓ *Coriobacteriaceae*, *Erysipelotrichaceae*, *Ruminococcaceae* (family level) *↓ Blautia* (genus level)
Kummen et al. [5]	Chronic HF	NA	*n* = 40 discovery *n* = 44 validation *n* = 266 control	16S rRNA	↓ -*Lachnospiraceae* family:
Cui et al. [58]	Stable chronic HF: Ischemic or dilated cardiomyopathy	58.1 ± 13.3 years	*n* = 53 HF *n* = 41 controls	16S rRNA	↑ *Ruminococcus gnavus* *↓ Faecalibacterium prausnitzii*
Beale et al. [70]	HFpEF	40–70 years	*n*= 26 HFpEF *n* = 67 control	16S rRNA	↓ *Ruminococcocus* spp.
Wang et al. [71]	Chronic HF	65 ± 3.17 years	*n* = 26 HF *n* = 26 controls	16S rRNA	*↑ Escherichia* *Shigella* *Ruminococcaceae*, *Lactobacillus* *Atopobium* *Romboutsia* *Streptococcus* *Haemophilus* *Klebsiella*
Katsimichas et al. [72]	Non-ischemic HFrEF	18–70	*n* = 28 HFrEF *n* = 19 controls	16S rRNA	*↑ Streptococcus* spp. *Veillonella* spp. ↓ SMB53
Hayashi et al. [48]	De novo acute decompensated HF/acute worsening of chronic HF	72 ± 18 years	*n* = 22 HF *n* = 11 controls	16S rRNA	*↑.Actinomycetota* phylum *Bifidobacterium* genus *↓.Megamonas* genus

**Table 3:** Known and proposed mechanisms by which the gut microbiota may influence cardiovascular drug outcomes, adapted from Tuteja et al. [121].

Drug	Bacteria	Mechanism(s)	Outcome
Known drug-microbiota interaction			
Digoxin [126]	*Eggerthella lenta*	Inactivation by reduction	Bacterial reductase activity reduces the quantity of active drug reaching target tissues
Proposed drug-microbiota interaction			
Simvastatin [127]	Not known	Microbial derived bile acids competing for host uptake transporters Disruption in bacterial communities with bile salt hydrolase (bsh) activity	Reduced amount of drug reaching target tissues FXR receptor signaling variability
Rosuvastatin [128]	Not known	Disruption in host gene expression of bile acid metabolism pathways Disruption in bacterial communities with bile salt hydrolase (bsh) activity	FXR receptor signaling variability
Atorvastatin [129]	Not known	Reduced quantity of secondary bile acids	FXR receptor signaling variability
Amlodipine [130]	Not known	Pre-systemic metabolism by dehydrogenation	Reduced quantity of active drug reaching target tissues
Captopril [121]	Not known	Not known	Improved villi length and reduced intestinal permeability
Aspirin [131]		Not known	Bacterial communities alteration
Warfarin [132]		Antibiotics eliminate vitamin K producing bacteria	Increased bleeding events

## References

[1] Groenewegen A., Rutten F.H., Mosterd A., Hoes A.W. (2020). Epidemiology of heart failure. Eur. J. Heart Fail..

[2] Tang W.H.W., Li D.Y., Hazen S.L. (2019). Dietary metabolism, the gut microbiome, and heart failure. Nat. Rev. Cardiol..

[3] Shirazi L.F., Bissett J., Romeo F., Mehta J.L. (2017). Role of Inflammation in Heart Failure. Curr. Atheroscler. Rep..

[4] Nemet I., Saha P.P., Gupta N., Zhu W., Romano K.A., Skye S.M., Cajka T., Mohan M.L., Li L., Wu Y. (2020). A Cardiovascular Disease-Linked Gut Microbial Metabolite Acts via Adrenergic Receptors. Cell.

[5] Kummen M., Mayerhofer C.C.K., Vestad B., Broch K., Awoyemi A., Storm-Larsen C., Ueland T., Yndestad A., Hov J.R., Trøseid M. (2018). Gut Microbiota Signature in Heart Failure Defined From Profiling of 2 Independent Cohorts. J. Am. Coll. Cardiol..

[6] Hietbrink F., Besselink M.G., Renooij W., de Smet M.B., Draisma A., van der Hoeven H., Pickkers P. (2009). Systemic inflammation increases intestinal permeability during experimental human endotoxemia. Shock.

[7] Mu F., Tang M., Guan Y., Lin R., Zhao M., Zhao J., Huang S., Zhang H., Wang J., Tang H. (2022). Knowledge Mapping of the Links Between the Gut Microbiota and Heart Failure: A Scientometric Investigation (2006–2021). Front. Cardiovasc. Med..

[8] Kamo T., Akazawa H., Suda W., Saga-Kamo A., Shimizu Y., Yagi H., Liu Q., Nomura S., Naito A.T., Takeda N. (2017). Dysbiosis and compositional alterations with aging in the gut microbiota of patients with heart failure. PLoS ONE.

[9] Sandek A., Bauditz J., Swidsinski A., Buhner S., Weber-Eibel J., von Haehling S., Schroedl W., Karhausen T., Doehner W., Rauchhaus M. (2007). Altered intestinal function in patients with chronic heart failure. J. Am. Coll. Cardiol..

[10] Pasini E., Aquilani R., Testa C., Baiardi P., Angioletti S., Boschi F., Verri M., Dioguardi F. (2016). Pathogenic Gut Flora in Patients With Chronic Heart Failure. JACC Heart Fail..

[11] Sun W., Du D., Fu T., Han Y., Li P., Ju H. (2022). Alterations of the Gut Microbiota in Patients With Severe Chronic Heart Failure. Front. Microbiol..

[12] Zhang Z., Cai B., Sun Y., Deng H., Wang H., Qiao Z. (2023). Alteration of the gut microbiota and metabolite phenylacetylglutamine in patients with severe chronic heart failure. Front. Cardiovasc. Med..

[13] Bozomitu L., Miron I., Adam Raileanu A., Lupu A., Paduraru G., Marcu F.M., Buga A.M.L., Rusu D.C., Dragan F., Lupu V.V. (2022). The Gut Microbiome and Its Implication in the Mucosal Digestive Disorders. Biomedicines.

[14] Di Tommaso N., Gasbarrini A., Ponziani F.R. (2021). Intestinal Barrier in Human Health and Disease. Int. J. Environ. Res. Public Health.

[15] Dekaboruah E., Suryavanshi M.V., Chettri D., Verma A.K. (2020). Human microbiome: An academic update on human body site specific surveillance and its possible role. Arch. Microbiol..

[16] Qin J., Li R., Raes J., Arumugam M., Burgdorf K.S., Manichanh C., Nielsen T., Pons N., Levenez F., Yamada T. (2010). A human gut microbial gene catalogue established by metagenomic sequencing. Nature.

[17] Arumugam M., Raes J., Pelletier E., Le Paslier D., Yamada T., Mende D.R., Fernandes G.R., Tap J., Bruls T., Batto J.M. (2011). Enterotypes of the human gut microbiome. Nature.

[18] Rinninella E., Raoul P., Cintoni M., Franceschi F., Miggiano G.A.D., Gasbarrini A., Mele M.C. (2019). What is the Healthy Gut Microbiota Composition? A Changing Ecosystem across Age, Environment, Diet, and Diseases. Microorganisms.

[19] Sharon I., Quijada N.M., Pasolli E., Fabbrini M., Vitali F., Agamennone V., Dötsch A., Selberherr E., Grau J.H., Meixner M. (2022). The Core Human Microbiome: Does It Exist and How Can We Find It? A Critical Review of the Concept. Nutrients.

[20] Sandek A., Bjarnason I., Volk H.D., Crane R., Meddings J.B., Niebauer J., Kalra P.R., Buhner S., Herrmann R., Springer J. (2012). Studies on bacterial endotoxin and intestinal absorption function in patients with chronic heart failure. Int. J. Cardiol..

[21] Piggott D.A., Tuddenham S. (2020). The gut microbiome and frailty. Transl. Res..

[22] Stinson L.F., Boyce M.C., Payne M.S., Keelan J.A. (2019). The Not-so-Sterile Womb: Evidence That the Human Fetus Is Exposed to Bacteria Prior to Birth. Front. Microbiol..

[23] Li S., Kararigas G. (2022). Role of Biological Sex in the Cardiovascular-Gut Microbiome Axis. Front. Cardiovasc. Med..

[24] Mills S., Stanton C., Lane J.A., Smith G.J., Ross R.P. (2019). Precision Nutrition and the Microbiome, Part I: Current State of the Science. Nutrients.

[25] Wang W., Zhu L.J., Leng Y.Q., Wang Y.W., Shi T., Wang W.Z., Sun J.C. (2023). Inflammatory Response: A Crucial Way for Gut Microbes to Regulate Cardiovascular Diseases. Nutrients.

[26] Steimle A., Frick J.S. (2016). Molecular Mechanisms of Induction of Tolerant and Tolerogenic Intestinal Dendritic Cells in Mice. J. Immunol. Res..

[27] Ilyas A., Wijayasinghe Y.S., Khan I. (2022). Implications of trimethylamine N-oxide (TMAO) and Betaine in Human Health: Beyond Being Osmoprotective Compounds. Front. Mol. Biosci..

[28] Su X., Gao Y., Yang R. (2022). Gut Microbiota-Derived Tryptophan Metabolites Maintain Gut and Systemic Homeostasis. Cells.

[29] Kamo T., Akazawa H., Suzuki J.I., Komuro I. (2017). Novel Concept of a Heart-Gut Axis in the Pathophysiology of Heart Failure. Korean Circ. J..

[30] Joice R., Yasuda K., Shafquat A., Morgan X.C., Huttenhower C. (2014). Determining microbial products and identifying molecular targets in the human microbiome. Cell Metab..

[31] Nagatomo Y., Tang W.H.W. (2015). Intersections between microbiome and heart failure: Revisiting the gut hypothesis. J. Card Fail..

[32] Gallo A., Macerola N., Favuzzi A.M., Nicolazzi M.A., Gasbarrini A., Montalto M. (2022). The Gut in Heart Failure: Current Knowledge and Novel Frontiers. Med. Princ. Pract..

[33] Sandek A., Swidsinski A., Schroedl W., Watson A., Valentova M., Herrmann R., Scherbakov N., Cramer L., Rauchhaus M., Grosse-Herrenthey A. (2014). Intestinal blood flow in patients with chronic heart failure: A link with bacterial growth, gastrointestinal symptoms, and cachexia. J. Am. Coll. Cardiol..

[34] Anker S.D., Egerer K.R., Volk H.D., Kox W.J., Poole-Wilson P.A., Coats A.J. (1997). Elevated soluble CD14 receptors and altered cytokines in chronic heart failure. Am. J. Cardiol..

[35] Lu Y.C., Yeh W.C., Ohashi P.S. (2008). LPS/TLR4 signal transduction pathway. Cytokine.

[36] Peschel T., Schonauer M., Thiele H., Anker S.D., Schuler G., Niebauer J. (2003). Invasive assessment of bacterial endotoxin and inflammatory cytokines in patients with acute heart failure. Eur. J. Heart Fail.

[37] Thierer J., Acosta A., Vainstein N., Sultan M., Francesia A., Marino J., Prado A.H., Guglielmone R., Trivi M., Boero L. (2010). Relation of left ventricular ejection fraction and functional capacity with metabolism and inflammation in chronic heart failure with reduced ejection fraction (from the MIMICA Study). Am. J. Cardiol..

[38] Deswal A., Petersen N.J., Feldman A.M., Young J.B., White B.G., Mann D.L. (2001). Cytokines and cytokine receptors in advanced heart failure: An analysis of the cytokine database from the Vesnarinone trial (VEST). Circulation.

[39] Rauchhaus M., Doehner W., Francis D.P., Davos C., Kemp M., Liebenthal C., Niebauer J., Hooper J., Volk H.D., Coats A.J. (2000). Plasma cytokine parameters and mortality in patients with chronic heart failure. Circulation.

[40] Kitai T., Kirsop J., Tang W.H. (2016). Exploring the Microbiome in Heart Failure. Curr. Heart Fail Rep..

[41] Chung E.S., Packer M., Lo K.H., Fasanmade A.A., Willerson J.T. (2003). Anti-TNF Therapy Against Congestive Heart Failure Investigators. Randomized, double-blind, placebo-controlled, pilot trial of infliximab, a chimeric monoclonal antibody to tumor necrosis factor-α, in patients with moderate-to-severe heart failure: Results of the anti-TNF Therapy Against Congestive Heart Failure (ATTACH) trial. Circulation.

[42] Mann D.L., McMurray J.J., Packer M., Swedberg K., Borer J.S., Colucci W.S., Djian J., Drexler H., Feldman A., Kober L. (2004). Targeted anticytokine therapy in patients with chronic heart failure: Results of the Randomized Etanercept Worldwide Evaluation (RENEWAL). Circulation.

[43] Yuzefpolskaya M., Bohn B., Nasiri M., Zuver A.M., Onat D.D., Royzman E.A., Nwokocha J., Mabasa M., Pinsino A., Brunjes D. (2020). Gut microbiota, endotoxemia, inflammation, and oxidative stress in patients with heart failure, left ventricular assist device, and transplant. J. Heart Lung Transplant..

[44] Al-Sadi R., Ye D., Boivin M., Guo S., Hashimi M., Ereifej L., Ma T.Y. (2014). Interleukin-6 modulation of intestinal epithelial tight junction permeability is mediated by JNK pathway activation of claudin-2 gene. PLoS ONE.

[45] Al-Sadi R.M., Ma T.Y. (2007). IL-1beta causes an increase in intestinal epithelial tight junction permeability. J. Immunol..

[46] Ma T.Y., Boivin M.A., Ye D., Pedram A., Said H.M. (2005). Mechanism of TNF-{alpha} modulation of Caco-2 intestinal epithelial tight junction barrier: Role of myosin light-chain kinase protein expression. Am. J. Physiol. Gastrointest. Liver Physiol..

[47] Al-Sadi R., Guo S., Ye D., Ma T.Y. (2013). TNF-alpha modulation of intestinal epithelial tight junction barrier is regulated by ERK1/2 activation of Elk-1. Am. J. Pathol..

[48] Hayashi T., Yamashita T., Watanabe H., Kami K., Yoshida N., Tabata T., Emoto T., Sasaki N., Mizoguchi T., Irino Y. (2018). Gut Microbiome and Plasma Microbiome-Related Metabolites in Patients With Decompensated and Compensated Heart Failure. Circ. J..

[49] Mamic P., Heidenreich P.A., Hedlin H., Tennakoon L., Staudenmayer K.L. (2016). Hospitalized Patients with Heart Failure and Common Bacterial Infections: A Nationwide Analysis of Concomitant Clostridium Difficile Infection Rates and In-Hospital Mortality. J. Card. Fail..

[50] Halaweish H.F., Boatman S., Staley C. (2022). Encapsulated Fecal Microbiota Transplantation: Development, Efficacy, and Clinical Application. Front. Cell Infect. Microbiol..

[51] Israr M.Z., Bernieh D., Salzano A., Cassambai S., Yazaki Y., Heaney L.M., Jones D.J.L., Ng L.L., Suzuki T. (2021). Association of gut-related metabolites with outcome in acute heart failure. Am. Heart J..

[52] Romano K.A., Nemet I., Prasad Saha P., Haghikia A., Li X.S., Mohan M.L., Lovano B., Castel L., Witkowski M., Buffa J.A. (2023). Gut Microbiota-Generated Phenylacetylglutamine and Heart Failure. Circ. Heart Fail..

[53] Guan X., Sun Z. (2023). The Role of Intestinal Flora and Its Metabolites in Heart Failure. Infect. Drug Resist..

[54] Zhang Y., Wang Y., Ke B., Du J. (2021). TMAO: How gut microbiota contributes to heart failure. Transl. Res..

[55] Spehlmann M.E., Rangrez A.Y., Dhotre D.P., Schmiedel N., Chavan N., Bang C., Müller O.J., Shouche Y.S., Franke A., Frank D. (2022). Heart Failure Severity Closely Correlates with Intestinal Dysbiosis and Subsequent Metabolomic Alterations. Biomedicines.

[56] Huang Z., Mei X., Jiang Y., Chen T., Zhou Y. (2022). Gut Microbiota in Heart Failure Patients With Preserved Ejection Fraction (GUMPTION Study). Front. Cardiovasc. Med..

[57] Luedde M., Winkler T., Heinsen F.A., Rühlemann M.C., Spehlmann M.E., Bajrovic A., Lieb W., Franke A., Ott S.J., Frey N. (2017). Heart failure is associated with depletion of core intestinal microbiota. ESC Heart Fail..

[58] Cui X., Ye L., Li J., Wang W., Li S., Bao M., Wu S., Li L., Geng B., Zhou X. (2018). Metagenomic and metabolomic analyses unveil dysbiosis of gut microbiota in chronic heart failure patients. Sci. Rep..

[59] Jia Q., Li H., Zhou H., Zhang X., Zhang A., Xie Y., Li Y., Lv S., Zhang J. (2019). Role and Effective Therapeutic Target of Gut Microbiota in Heart Failure. Cardiovasc. Ther..

[60] Li L., Zhong S.J., Hu S.Y., Cheng B., Qiu H., Hu Z.X. (2021). Changes of gut microbiome composition and metabolites associated with hypertensive heart failure rats. BMC Microbiol..

[61] Zhu Q., Gao R., Zhang Y., Pan D., Zhu Y., Zhang X., Yang R., Jiang R., Xu Y., Qin H. (2018). Dysbiosis signatures of gut microbiota in coronary artery disease. Physiol Genom..

[62] Duncan S.H., Holtrop G., Lobley G.E., Calder A.G., Stewart C.S., Flint H.J. (2004). Contribution of acetate to butyrate formation by human faecal bacteria. Br. J. Nutr..

[63] Sokol H., Pigneur B., Watterlot L., Lakhdari O., Bermudez-Humaran L.G., Gratadoux J.J., Blugeon S., Bridonneau C., Furet J.P., Corthier G. (2008). Faecalibacterium prausnitzii is an anti-inflammatory commensal bacterium identified by gut microbiota analysis of Crohn disease patients. Proc. Natl. Acad. Sci. USA.

[64] Martin R., Miquel S., Chain F., Natividad J.M., Jury J., Lu J., Sokol H., Theodorou V., Bercik P., Verdu E.F. (2015). Faecalibacterium prausnitzii prevents physiological damages in a chronic low-grade inflammation murine model. BMC Microbiol..

[65] Leonel A.J., Alvarez-Leite J.I. (2012). Butyrate: Implications for intestinal function. Curr. Opin. Clin. Nutr. Metab. Care.

[66] Furusawa Y., Obata Y., Fukuda S., Endo T.A., Nakato G., Takahashi D., Nakanishi Y., Uetake C., Kato K., Kato T. (2013). Commensal microbe-derived butyrate induces the differentiation of colonic regulatory T cells. Nature.

[67] Mayerhofer C.C.K., Kummen M., Holm K., Broch K., Awoyemi A., Vestad B., Storm-Larsen C., Seljeflot I., Ueland T., Bohov P. (2020). Low fibre intake is associated with gut microbiota alterations in chronic heart failure. ESC Heart Fail..

[68] Engels C., Ruscheweyh H.J., Beerenwinkel N., Lacroix C., Schwab C. (2016). The Common Gut Microbe Eubacterium hallii also Contributes to Intestinal Propionate Formation. Front. Microbiol..

[69] Zhou B., Yuan Y., Zhang S., Guo C., Li X., Li G., Xiong W., Zeng Z. (2020). Intestinal Flora and Disease Mutually Shape the Regional Immune System in the Intestinal Tract. Front. Immunol..

[70] Beale A.L., O’Donnell J.A., Nakai M.E., Nanayakkara S., Vizi D., Carter K., Dean E., Ribeiro R.V., Yiallourou S., Carrington M.J. (2021). The Gut Microbiome of Heart Failure With Preserved Ejection Fraction. J. Am. Heart Assoc..

[71] Wang Z., Cai Z., Ferrari M.W., Liu Y., Li C., Zhang T., Lyu G. (2021). The Correlation between Gut Microbiota and Serum Metabolomic in Elderly Patients with Chronic Heart Failure. Mediat. Inflamm..

[72] Katsimichas T., Ohtani T., Motooka D., Tsukamoto Y., Kioka H., Nakamoto K., Konishi S., Chimura M., Sengoku K., Miyawaki H. (2018). Non-Ischemic Heart Failure With Reduced Ejection Fraction Is Associated With Altered Intestinal Microbiota. Circ. J..

[73] Murphy S.P., Ibrahim N.E., Januzzi J.L. (2020). Heart failure with reduced ejection fraction. JAMA.

[74] Wu J., Zheng H., Liu X., Chen P., Zhang Y., Luo J., Kuang J., Li J., Yang Y., Ma T. (2020). Prognostic value of secreted frizzled-related protein 5 in heart failure patients with and without type 2 diabetes mellitus. Circ. Heart Fail..

[75] Rodrigues A., Gonçalves A., Morais J., Araujo R., Falcão-Pires I. (2023). Diet-Induced Microbiome’s Impact on Heart Failure: A Double-Edged Sword. Nutrients.

[76] Shrivastava A., Haase T., Zeller T., Schulte C. (2020). Biomarkers for Heart Failure Prognosis: Proteins, Genetic Scores and Non-coding RNAs. Front. Cardiovasc. Med..

[77] Yu W., Jiang Y., Xu H., Zhou Y. (2023). The Interaction of Gut Microbiota and Heart Failure with Preserved Ejection Fraction: From Mechanism to Potential Therapies. Biomedicines.

[78] Hairrman R.S., Gouveia C.G., Sichinel Â.H., Silva L.S.A., Oliveira T.S.S., Farias M.N. (2021). Tmao and the relationship with cardiovascular disease: The elderly and their physiological aspects. Braz. J. Dev..

[79] Trøseid M., Andersen G.Ø., Broch K., Hov J.R. (2020). The gut microbiome in coronary artery disease and heart failure: Current knowledge and future directions. EBioMedicine.

[80] Tang W.H., Wang Z., Fan Y., Levison B., Hazen J.E., Donahue L.M., Wu Y., Hazen S.L. (2014). Prognostic value of elevated levels of intestinal microbe-generated metabolite trimethylamine-N-oxide in patients with heart failure: Refining the gut hypothesis. J. Am. Coll. Cardiol..

[81] Savji N., Meijers W.C., Bartz T.M., Bhambhani V., Cushman M., Nayor M., Kizer J.R., Sarma A., Blaha M.J., Gansevoort R.T. (2018). The Association of Obesity and Cardiometabolic Traits With Incident HFpEF and HFrEF. JACC Heart Fail..

[82] Paulus W.J., Tschöpe C. (2013). A novel paradigm for heart failure with preserved ejection fraction: Comorbidities drive myocardial dysfunction and remodeling through coronary microvascular endothelial inflammation. J. Am. Coll. Cardiol..

[83] Wang Y.C., Liang C.S., Gopal D.M., Ayalon N., Donohue C., Santhanakrishnan R., Sandhu H., Perez A.J., Downing J., Gokce N. (2015). Preclinical Systolic and Diastolic Dysfunctions in Metabolically Healthy and Unhealthy Obese Individuals. Circ. Heart Fail..

[84] Khan M.F., Movahed M.R. (2013). Obesity cardiomyopathy and systolic function: Obesity is not independently associated with dilated cardiomyopathy. Heart Fail. Rev..

[85] Hildebrandt M.A., Hoffmann C., Sherrill-Mix S.A., Keilbaugh S.A., Hamady M., Chen Y.Y., Knight R., Ahima R.S., Bushman F., Wu G.D. (2009). High-fat diet determines the composition of the murine gut microbiome independently of obesity. Gastroenterology.

[86] Ley R.E., Turnbaugh P.J., Klein S., Gordon J.I. (2006). Microbial ecology: Human gut microbes associated with obesity. Nature.

[87] Kriaa A., Bourgin M., Potiron A., Mkaouar H., Jablaoui A., Gérard P., Maguin E., Rhimi M. (2019). Microbial impact on cholesterol and bile acid metabolism: Current status and future prospects. J. Lipid Res..

[88] Zhong H., Ren H., Lu Y., Fang C., Hou G., Yang Z., Chen B., Yang F., Zhao Y., Shi Z. (2019). Distinct gut metagenomics and metaproteomics signatures in prediabetics and treatment-naïve type 2 diabetics. EBioMedicine.

[89] Gurung M., Li Z., You H., Rodrigues R., Jump D.B., Morgun A., Shulzhenko N. (2020). Role of gut microbiota in type 2 diabetes pathophysiology. EBioMedicine.

[90] Sanna S., van Zuydam N.R., Mahajan A., Kurilshikov A., Vich Vila A., Võsa U., Mujagic Z., Masclee A.A.M., Jonkers D.M.A.E., Oosting M. (2019). Causal relationships among the gut microbiome, short-chain fatty acids and metabolic diseases. Nat. Genet..

[91] Qin J., Li Y., Cai Z., Li S., Zhu J., Zhang F., Liang S., Zhang W., Guan Y., Shen D. (2012). A metagenome-wide association study of gut microbiota in type 2 diabetes. Nature.

[92] Li Q., Chang Y., Zhang K., Chen H., Tao S., Zhang Z. (2020). Implication of the gut microbiome composition of type 2 diabetic patients from northern China. Sci. Rep..

[93] Okamura M., Ueno T., Tanaka S., Murata Y., Kobayashi H., Miyamoto A., Abe M., Fukuda N. (2021). Increased expression of acyl-CoA oxidase 2 in the kidney with plasma phytanic acid and altered gut microbiota in spontaneously hypertensive rats. Hypertens Res..

[94] Yang T., Santisteban M.M., Rodriguez V., Li E., Ahmari N., Carvajal J.M., Zadeh M., Gong M., Qi Y., Zubcevic J. (2015). Gut dysbiosis is linked to hypertension. Hypertension.

[95] Adnan S., Nelson J.W., Ajami N.J., Venna V.R., Petrosino J.F., Bryan R.M., Durgan D.J. (2017). Alterations in the gut microbiota can elicit hypertension in rats. Physiol. Genom..

[96] McDonagh T.A., Metra M., Adamo M., Gardner R.S., Baumbach A., Böhm M., Burri H., Butler J., Čelutkienė J., Chioncel O. (2021). ESC Guidelines for the diagnosis and treatment of acute and chronic heart failure. Eur. Heart J..

[97] Heidenreich P.A., Bozkurt B., Aguilar D., Allen L.A., Byun J.J., Colvin M.M., Deswal A., Drazner M.H., Dunlay S.M., Evers L.R. (2022). AHA/ACC/HFSA Guideline for the Management of Heart Failure: A Report of the American College of Cardiology/American Heart Association Joint Committee on Clinical Practice Guidelines. Circulation.

[98] Verhaar B.J.H., Prodan A., Nieuwdorp M., Muller M. (2020). Gut Microbiota in Hypertension and Atherosclerosis: A Review. Nutrients.

[99] Liu B.N., Liu X.T., Liang Z.H., Wang J.H. (2021). Gut microbiota in obesity. World J. Gastroenterol..

[100] Hobby G.P., Karaduta O., Dusio G.F., Singh M., Zybailov B.L., Arthur J.M. (2019). Chronic kidney disease and the gut microbiome. Am. J. Physiol. Renal. Physiol..

[101] Francisqueti-Ferron F.V., Nakandakare-Maia E.T., Siqueira J.S., Ferron A.J.T., Vieira T.A., Bazan S.G.Z., Corrêa C.R. (2022). The role of gut dysbiosis-associated inflammation in heart failure. Rev. Assoc. Med. Bras.

[102] Tuerhongjiang G., Guo M., Qiao X., Lou B., Wang C., Wu H., Wu Y., Yuan Z., She J. (2021). Interplay Between Gut Microbiota and Amino Acid Metabolism in Heart Failure. Front. Cardiovasc. Med..

[103] Chen W.S., Wang C.H., Cheng C.W., Liu M.H., Chu C.M., Wu H.P., Huang P.C., Lin Y.T., Ko T., Chen W.H. (2020). Elevated plasma phenylalanine predicts mortality in critical patients with heart failure. ESC Heart Fail..

[104] Delles C., Rankin N.J., Boachie C., McConnachie A., Ford I., Kangas A., Soininen P., Trompet S., Mooijaart S.P., Jukema J.W. (2018). Nuclear magnetic resonance-based metabolomics identifies phenylalanine as a novel predictor of incident heart failure hospitalisation: Results from PROSPER and FINRISK 1997. Eur. J. Heart Fail..

[105] Hayashi T., Yamashita T., Takahashi T., Tabata T., Watanabe H., Gotoh Y., Shinohara M., Kami K., Tanaka H., Matsumoto K. (2021). Uncovering the Role of Gut Microbiota in Amino Acid Metabolic Disturbances in Heart Failure Through Metagenomic Analysis. Front. Cardiovasc. Med..

[106] Chen X., Li H.Y., Hu X.M., Zhang Y., Zhang S.Y. (2019). Current understanding of gut microbiota alterations and related therapeutic intervention strategies in heart failure. Chin. Med. J..

[107] Koeth R.A., Wang Z., Levison B.S., Buffa J., Org E., Sheehy B., Britt E., Fu X., Wu Y., Li L. (2013). Intestinal microbiota metabolism of L-carnitine, a nutrient in red meat, promotes atherosclerosis. Nat. Med..

[108] Wang Z., Roberts A.B., Buffa J.A., Levison B.S., Zhu W., Org E., Gu X., Huang Y., Zamanian-Daryoush M., Culley M.K. (2015). Non-lethal Inhibition of Gut Microbial Trimethylamine Production for the Treatment of Atherosclerosis. Cell.

[109] Yang W., Zhang S., Zhu J., Jiang H., Jia D., Ou T., Qi Z., Zou Y., Qian J., Sun A. (2019). Gut microbe-derived metabolite trimethylamine N-oxide accelerates fibroblast-myofibroblast differentiation and induces cardiac fibrosis. J. Mol. Cell. Cardiol..

[110] Hinderer S., Schenke-Layland K. (2019). Cardiac fibrosis—A short review of causes and therapeutic strategies. Adv. Drug Deliv. Rev..

[111] Liu Y., Dai M. (2020). Trimethylamine N-Oxide Generated by the Gut Microbiota Is Associated with Vascular Inflammation: New Insights into Atherosclerosis. Mediat. Inflamm..

[112] Salzano A., Cassambai S., Yazaki Y., Israr M.Z., Bernieh D., Wong M., Suzuki T. (2022). The Gut Axis Involvement in Heart Failure: Focus on Trimethylamine N-oxide. Cardiol. Clin..

[113] Xu J., Yang Y. (2021). Gut microbiome and its meta-omics perspectives: Profound implications for cardiovascular diseases. Gut Microbes..

[114] Suzuki T., Heaney L.M., Bhandari S.S., Jones D.J., Ng L.L. (2016). Trimethylamine N-oxide and prognosis in acute heart failure. Heart.

[115] Dong Z., Zheng S., Shen Z., Luo Y., Hai X. (2021). Trimethylamine N-Oxide is Associated with Heart Failure Risk in Patients with Preserved Ejection Fraction. Lab. Med..

[116] Cuesta-Zuluaga J., Mueller N.T., Álvarez-Quintero R., Velásquez-Mejía E.P., Sierra J.A., Corral es-Agudelo V., Carmona J.A., Abad J.M., Escobar J.S. (2018). Higher fecal short-chain fatty acid levels are associated with gut microbiome dysbiosis, obesity, hypertension and cardiometabolic disease risk factors. Nutrients.

[117] Aguilar E.C., Santos L.C., Leonel A.J., de Oliveira J.S., Santos E.A., Navia-Pelaez J.M., da Silva J.F., Mendes B.P., Capettini L.S., Teixeira L.G. (2016). Oral butyrate reduces oxidative stress in atherosclerotic lesion sites by a mechanism involving NADPH oxidase down-regulation in endothelial cells. J. Nutr. Biochem..

[118] Aguilar E.C., Leonel A.J., Teixeira L.G., Silva A.R., Silva J.F., Pelaez J.M., Capettini L.S., Lemos V.S., Santos R.A., Alvarez-Leite J.I. (2014). Butyrate impairs atherogenesis by reducing plaque inflammation and vulnerability and decreasing NFκB activation. Nutr. Metab. Cardiovasc. Dis..

[119] Wu J., Qiu M., Sun L., Wen J., Liang D.L., Zheng S., Huang Y. (2022). α-Linolenic Acid and Risk of Heart Failure: A Meta-Analysis. Front. Cardiovasc. Med..

[120] Luo Q., Hu Y., Chen X., Luo Y., Chen J., Wang H. (2022). Effects of Gut Microbiota and Metabolites on Heart Failure and Its Risk Factors: A Two-Sample Mendelian Randomization Study. Front. Nutr..

[121] Tuteja S., Ferguson J.F. (2019). Gut Microbiome and Response to Cardiovascular Drugs. Circ. Genom. Precis Med..

[122] Kim D.-H. (2015). Gut microbiota-mediated drug-antibiotic interactions. Drug Metab. Dispos..

[123] Sousa T., Paterson R., Moore V., Carlsson A., Abrahamsson B., Basit A.W. (2008). The gastrointestinal microbiota as a site for the biotransformation of drugs. Int. J. Pharm..

[124] Zhernakova A., Kurilshikov A., Bonder M.J., Tigchelaar E.F., Schirmer M., Vatanen T., Mujagic Z., Vila A.V., Falony G., Vieira-Silva S. (2016). Population-based metagenomics analysis reveals markers for gut microbiome composition and diversity. Science.

[125] Jackson M.A., Verdi S., Maxan M.E., Shin C.M., Zierer J., Bowyer R.C.E., Martin T., Williams F.M.K., Menni C., Bell J.T. (2018). Gut microbiota associations with common diseases and prescription medications in a population-based cohort. Nat. Commun..

[126] Haiser H.J., Gootenberg D.B., Chatman K., Sirasani G., Balskus E.P., Turnbaugh P.J. (2013). Predicting and manipulating cardiac drug inactivation by the human gut bacterium eggerthella lenta. Science.

[127] Dias A.M., Cordeiro G., Estevinho M.M., Veiga R., Figueira L., Reina-Couto M., Magro F., The Clinical Pharmacology Unit, São João Hospital University Centre (2020). Gut bacterial microbiome composition and statin intake-A systematic review. Pharmacol. Res. Perspect..

[128] Liu Y., Song X., Zhou H., Zhou X., Xia Y., Dong X., Zhong W., Tang S., Wang L., Wen S. (2018). Gut microbiome associates with lipid-lowering effect of rosuvastatin in vivo. Front. Microbiol..

[129] Fu Z.D., Cui J.Y., Klaassen C.D. (2014). Atorvastatin induces bile acid-synthetic enzyme cyp7a1 by suppressing fxr signaling in both liver and intestine in mice. J. Lipid. Res..

[130] Yoo H.H., Kim I.S., Yoo D.H., Kim D.H. (2016). Effects of orally administered antibiotics on the bioavailability of amlodipine: Gut microbiota-mediated drug interaction. J. Hypertens.

[131] Alhajri N., Khursheed R., Ali M.T., Abu Izneid T., Al-Kabbani O., Al-Haidar M.B., Al-Hemeiri F., Alhashmi M., Pottoo F.H. (2021). Cardiovascular Health and The Intestinal Microbial Ecosystem: The Impact of Cardiovascular Therapies on The Gut Microbiota. Microorganisms.

[132] Holbrook A.M., Pereira J.A., Labiris R., McDonald H., Douketis J.D., Crowther M., Wells P.S. (2005). Systematic overview of warfarin and its drug and food interactions. Arch. Intern. Med..

[133] Saha J.R., Butler V.P., Neu H.C., Lindenbaum J. (1983). Digoxin-inactivating bacteria: Identification in human gut flora. Science.

[134] Lindenbaum J., Rund D.G., Butler V.P., Tse-Eng D., Saha J.R. (1981). Inactivation of digoxin by the gut flora: Reversal by antibiotic therapy. N. Engl. J. Med..

[135] Kim I.S., Yoo D.H., Jung I.H., Lim S., Jeong J.J., Kim K.A., Bae O.N., Yoo H.H., Kim D.H. (2016). Reduced metabolic activity of gut microbiota by antibiotics can potentiate the antithrombotic effect of aspirin. Biochem. Pharmacol..

[136] Mortensen B., Murphy C., O’Grady J., Lucey M., Elsafi G., Barry L., Westphal V., Wellejus A., Lukjancenko O., Eklund A. (2019). Bifidobacteriumbreve Bif195 Protects Against Small-Intestinal Damage Caused by Acetylsalicylic Acid in Healthy Volunteers. Gastroenterology.

[137] Lane M.A., Zeringue A., McDonald J.R. (2014). Serious bleeding events due to warfarin and antibiotic co-prescription in a cohort of veterans. Am. J. Med..

[138] Shearer M.J., Newman P. (2008). Metabolism and cell biology of vitamin k. Thromb. Haemost..

[139] Xu H., Wang X., Feng W., Liu Q., Zhou S., Liu Q., Cai L. (2020). The gut microbiota and its interactions with cardiovascular disease. Microb. Biotechnol..

[140] Khan T.J., Ahmed Y.M., Zamzami M.A., Siddiqui A.M., Khan I., Baothman O.A.S., Mehanna M.G., Kuerban A., Kaleemuddin M., Yasir M. (2018). Atorvastatin treatment modulates the gut microbiota of the hypercholesterolemic patients. Omics J. Integr. Biol..

[141] Jones M.L., Martoni C.J., Prakash S. (2012). Cholesterol lowering and inhibition of sterol absorption by lactobacillus reuteri ncimb 30242: A randomized controlled trial. Eur. J. Clin. Nutr..

[142] Jones B.V., Begley M., Hill C., Gahan C.G., Marchesi J.R. (2008). Functional and comparative metagenomic analysis of bile salt hydrolase activity in the human gut microbiome. Proc. Natl. Acad. Sci. USA.

[143] Khan T.J., Ahmed Y.M., Zamzami M.A., Mohamed S.A., Khan I., Baothman O.A.S., Mehanna M.G., Yasir M. (2018). Effect of atorvastatin on the gut microbiota of high fat diet-induced hypercholesterolemic rats. Sci. Rep..

[144] Li M., Al-Sarraf A., Sinclair G., Frohlich J. (2011). Fish odour syndrome. Cmaj.

[145] David L.A., Maurice C.F., Carmody R.N., Gootenberg D.B., Button J.E., Wolfe B.E., Ling A.V., Devlin A.S., Varma Y., Fischbach M.A. (2014). Diet rapidly and reproducibly alters the human gut microbiome. Nature.

[146] Merra G., Noce A., Marrone G., Cintoni M., Tarsitano M.G., Capacci A., De Lorenzo A. (2020). Influence of Mediterranean Diet on Human Gut Microbiota. Nutrients.

[147] De Filippis F., Pellegrini N., Vannini L., Jeffery I.B., La Storia A., Laghi L., Serrazanetti D.I., Di Cagno R., Ferrocino I., Lazzi C. (2016). High-level adherence to a Mediterranean diet beneficially impacts the gut microbiota and associated metabolome. Gut.

[148] Lopez-Garcia E., Rodriguez-Artalejo F., Li T.Y., Fung T.T., Li S., Willett W.C., Rimm E.B., Hu F.B. (2014). The Mediterranean-style dietary pattern and mortality among men and women with cardiovascular disease. Am. J. Clin. Nutr..

[149] Kouvari M., Chrysohoou C., Aggelopoulos P., Tsiamis E., Tsioufis K., Pitsavos C., Tousoulis D. (2017). Mediterranean diet and prognosis of first-diagnosed Acute Coronary Syndrome patients according to heart failure phenotype: Hellenic Heart Failure Study. Eur. J. Clin. Nutr..

[150] Mamic P., Chaikijurajai T., Tang W.H.W. (2021). Gut microbiome—A potential mediator of pathogenesis in heart failure and its comorbidities: State-of-the-art review. J. Mol. Cell Cardiol..

[151] Salehi-Abargouei A., Maghsoudi Z., Shirani F., Azadbakht L. (2013). Effects of Dietary Approaches to Stop Hypertension (DASH)-style diet on fatal or nonfatal cardiovascular diseases—Incidence: A systematic review and meta-analysis on observational prospective studies. Nutrition.

[152] Levitan E.B., Wolk A., Mittleman M.A. (2009). Consistency with the DASH diet and incidence of heart failure. Arch. Intern. Med..

[153] Marques F.Z., Nelson E., Chu P.Y., Horlock D., Fiedler A., Ziemann M., Tan J.K., Kuruppu S., Rajapakse N.W., El-Osta A. (2017). High-Fiber diet and acetate supplementation change the gut microbiota and prevent the development of hypertension and heart failure in hypertensive mice. Circulation.

[154] Food and Agriculture Organization (FAO) of the United Nations Food Safety and Quality: Probiotics. http://www.fao.org/food/food-safety-quality/a-z-index/probiotics/en/.

[155] Kang H.J., Im S.H. (2015). Probiotics as an Immune Modulator. J. Nutr. Sci. Vitaminol..

[156] Hacini-Rachinel F., Gheit H., Le Luduec J.B., Dif F., Nancey S., Kaiserlian D. (2009). Oral probiotic control skin inflammation by acting on both effector and regulatory T cells. PLoS ONE.

[157] Ganesh B.P., Versalovic J. (2015). Luminal Conversion and Immunoregulation by Probiotics. Front. Pharmacol..

[158] Zhao X., Zhang Z., Hu B., Huang W., Yuan C., Zou L. (2018). Response of gut microbiota to metabolite changes induced by endurance exercise. Front. Microbiol..

[159] Gan X.T., Ettinger G., Huang C.X., Burton J.P., Haist J.V., Rajapurohitam V., Sidaway J.E., Martin G., Gloor G.B., Swann J.R. (2014). Probiotic administration attenuates myocardial hypertrophy and heart failure after myocardial infarction in the rat. Circ. Heart Fail..

[160] Lin P.P., Hsieh Y.M., Kuo W.W., Lin Y.M., Yeh Y.L., Lin C.C., Tsai F.J., Tsai C.H., Huang C.Y., Tsai C.C. (2013). Probiotic-fermented purple sweet potato yogurt activates compensatory IGF IR/PI3K/Akt survival pathways and attenuates cardiac apoptosis in the hearts of spontaneously hypertensive rats. Int. J. Mol. Med..

[161] Li K., Tian P., Wang S., Lei P., Qu L., Huang J., Shan J., Li B. (2017). Targeting gut microbiota: Lactobacillus alleviated type 2 diabetes via inhibiting LPS secretion and activating GPR43 pathway. J. Funct. Foods.

[162] Wang X., Zhang M., Chen S., Sun Y., Ren F., Tong Q. (2017). Effects of Lactobacillus paracasei L9 on the content of short-chain fatty acids in the intestine of mice. Food Sci..

[163] Ettinger G., MacDonald K., Reid G., Burton J.P. (2014). The influence of the human microbiome and probiotics on cardiovascular health. Gut Microbes.

[164] Costanza A.C., Moscavitch S.D., Faria Neto H.C., Mesquita E.T. (2015). Probiotic therapy with Saccharomyces boulardii for heart failure patients: A randomized, double-blind, placebo-controlled pilot trial. Int. J. Cardiol..

[165] Awoyemi A., Mayerhofer C., Felix A.S., Hov J.R., Moscavitch S.D., Lappegård K.T., Hovland A., Halvorsen S., Halvorsen B., Gregersen I. (2021). Rifaximin or Saccharomyces boulardii in heart failure with reduced ejection fraction: Results from the randomized GutHeart trial. EBioMedicine.

[166] Lam V., Su J., Koprowski S., Hsu A., Tweddell J.S., Rafiee P., Gross G.J., Salzman N.H., Baker J.E. (2012). Intestinal microbiota determine severity of myocardial infarction in rats. FASEB J..

[167] Ponziani F.R., Zocco M.A., D’Aversa F., Pompili M., Gasbarrini A. (2017). Eubiotic properties of rifaximin: Disruption of the traditional concepts in gut microbiota modulation. World J. Gastroenterol..

[168] Chen M.L., Yi L., Zhang Y., Zhou X., Ran L., Yang J., Zhu J.D., Zhang Q.Y., Mi M.T. (2016). Resveratrol Attenuates Trimethylamine-N-Oxide (TMAO)-Induced Atherosclerosis by Regulating TMAO Synthesis and Bile Acid Metabolism via Remodeling of the Gut Microbiota. mBio.

[169] Conraads V.M., Jorens P.G., De Clerck L.S., Van Saene H.K., Ieven M.M., Bosmans J.M., Schuerwegh A., Bridts C.H., Wuyts F., Stevens W.J. (2004). Selective intestinal decontamination in advanced chronic heart failure: A pilot trial. Eur. J. Heart Fail..

[170] Kuehn B.M. (2019). Gut Microbes Role in Heart Failure Explored. Circulation.

[171] Davani-Davari D., Negahdaripour M., Karimzadeh I., Seifan M., Mohkam M., Masoumi S.J., Berenjian A., Ghasemi Y. (2019). Prebiotics: Definition, Types, Sources, Mechanisms, and Clinical Applications. Foods.

[172] Miao T., Yu Y., Sun J., Ma A., Yu J., Cui M., Yang L., Wang H. (2021). Decrease in abundance of bacteria of the genus Bifidobacterium in gut microbiota may be related to pre-eclampsia progression in women from East China. Food Nutr. Res..

[173] Kumar S.A., Ward L.C., Brown L. (2016). Inulin oligofructose attenuates metabolic syndrome in high-carbohydrate, high-fat diet-fed rats. Br. J. Nutr..

[174] Salles B.I.M., Cioffi D., Ferreira S.R.G. (2020). Probiotics supplementation and insulin resistance: A systematic review. Diabetol. Metab. Syndr..

[175] Chen K., Zheng X., Feng M., Li D., Zhang H. (2017). Gut Microbiota-Dependent Metabolite Trimethylamine N-Oxide Contributes to Cardiac Dysfunction in Western Diet-Induced Obese Mice. Front. Physiol..

[176] Organ C.L., Li Z., Sharp T.E., Polhemus D.J., Gupta N., Goodchild T.T., Tang W.H.W., Hazen S.L., Lefer D.J. (2020). Inhibition of Gut Microbial Trimethylamine N-oxide Production Improves Cardiac Function and Remodeling in a Murine Modelof Heart Failure. J. Am. Heart Assoc..

[177] Allegretti J.R., Mullish B.H., Kelly C., Fischer M. (2019). The evolution of the use of faecal microbiota transplantation and emerging therapeutic indications. Lancet.

[178] Ghani R., Mullish B.H., McDonald J.A.K., Ghazy A., Williams H.R.T., Brannigan E.T., Mookerjee S., Satta G., Gilchrist M., Duncan N. (2021). Disease Prevention Not Decolonization: A Model for Fecal Microbiota Transplantation in Patients Colonized With Multidrug-resistant Organisms. Clin. Infect. Dis..

[179] Ianiro G., Eusebi L.H., Black C.J., Gasbarrini A., Cammarota G., Ford A.C. (2019). Systematic review with meta-analysis: Efficacy of faecal microbiota transplantation for the treatment of irritable bowel syndrome. Aliment. Pharmacol. Ther..

[180] Bajaj J.S., Salzman N.H., Acharya C., Sterling R.K., White M.B., Gavis E.A., Fagan A., Hayward M., Holtz M.L., Matherly S. (2019). Fecal Microbial Transplant Capsules Are Safe in Hepatic Encephalopathy: A Phase 1, Randomized, Placebo-Controlled Trial. Hepatology.

[181] Buffie C.G., Bucci V., Stein R.R., McKenney P.T., Ling L., Gobourne A., No D., Liu H., Kinnebrew M., Viale A. (2014). Precision microbiome reconstitution restores bile acid mediated resistance to Clostridium difficile. Nature.

[182] Ghani R., Mullish B.H., Roberts L.A., Davies F.J., Marchesi J.R. (2022). The potential utility of fecal (or intestinal) microbiota transplantation in controlling infectious diseases. Gut Microbes.

[183] Dias C.K., Starke R., Pylro V.S., Morais D.K. (2020). Database limitations for studying the human gut microbiome. PeerJ Comput. Sci..

[184] Inkpen S.A., Douglas G.M., Brunet T.D.P., Leuschen K., Doolittle W.F., Langille M.G.I. (2017). The coupling of taxonomy and function in microbiomes. Biol. Philos..

[185] Cassotta M., Forbes-Hernández T.Y., Calderón Iglesias R., Ruiz R., Elexpuru Zabaleta M., Giampieri F., Battino M. (2020). Links between Nutrition, Infectious Diseases, and Microbiota: Emerging Technologies and Opportunities for Human-Focused Research. Nutrients.

[186] Triposkiadis F., Xanthopoulos A., Parissis J., Butler J., Farmakis D. (2022). Pathogenesis of chronic heart failure: Cardiovascular aging, risk factors, comorbidities, and disease modifiers. Heart Fail. Rev..

